# Preclinical evaluation of CDK4 phosphorylation predicts high sensitivity of pleural mesotheliomas to CDK4/6 inhibition

**DOI:** 10.1002/1878-0261.13351

**Published:** 2022-12-19

**Authors:** Sabine Paternot, Eric Raspé, Clément Meiller, Maxime Tarabichi, Jean‐Baptiste Assié, Frederick Libert, Myriam Remmelink, Xavier Bisteau, Patrick Pauwels, Yuna Blum, Nolwenn Le Stang, Séverine Tabone‐Eglinger, Françoise Galateau‐Sallé, Christophe Blanquart, Jan P. Van Meerbeeck, Thierry Berghmans, Didier Jean, Pierre P. Roger

**Affiliations:** ^1^ Institut de Recherche Interdisciplinaire en Biologie Humaine et Moléculaire (IRIBHM) Université Libre de Bruxelles Belgium; ^2^ ULB‐Cancer Research Center (U‐CRC) Université Libre de Bruxelles Belgium; ^3^ Université de Paris Centre de Recherche des Cordeliers, Inserm, Sorbonne Université, Functional Genomics of Solid Tumors France; ^4^ CEpiA (Clinical Epidemiology and Ageing), EA 7376‐IMRB University Paris‐Est Créteil France; ^5^ GRC OncoThoParisEst, Service de Pneumologie, CHI Créteil, UPEC Créteil France; ^6^ BRIGHTCore, ULB Brussels Belgium; ^7^ Department of Pathology, Erasme Hospital Université Libre de Bruxelles Belgium; ^8^ Center for Oncological Research (CORE) Integrated Personalized and Precision Oncology Network (IPPON) Wilrijk Belgium; ^9^ Department of Pathology Antwerp University Hospital Edegem Belgium; ^10^ Programme Cartes d'Identité des Tumeurs (CIT), Ligue Nationale Contre Le Cancer Paris France; ^11^ MESOBANK, Department of Biopathology, Centre Léon Bérard Lyon France; ^12^ Cancer Research Center INSERM U1052‐CNRS 5286R Lyon France; ^13^ Inserm, CNRS, Université d'Angers, CRCI2NA Nantes Université Nantes France; ^14^ Department of Thoracic Oncology Antwerp University Hospital Edegem Belgium; ^15^ Clinic of Thoracic Oncology Institut Jules Bordet, Université Libre de Bruxelles Brussels Belgium; ^16^ Present address: IGDR UMR 6290, CNRS, Université de Rennes 1 France

**Keywords:** biomarker, CDK4 Thr172‐phosphorylation, cell cycle, mesothelioma, palbociclib, senescence‐associated secretory phenotype

## Abstract

Malignant pleural mesothelioma (MPM) is an aggressive cancer with limited therapeutic options. We evaluated the impact of CDK4/6 inhibition by palbociclib in 28 MPM cell lines including 19 patient‐derived ones, using various approaches including RNA‐sequencing. Palbociclib strongly and durably inhibited the proliferation of 23 cell lines, indicating a unique sensitivity of MPM to CDK4/6 inhibition. When observed, insensitivity to palbociclib was mostly explained by the lack of active T172‐phosphorylated CDK4. This was associated with high p16^INK4A^ (*CDKN2A*) levels that accompany *RB1* defects or inactivation, or (unexpectedly) *CCNE1* overexpression in the presence of wild‐type *RB1*. Prolonged palbociclib treatment irreversibly inhibited proliferation despite re‐induction of cell cycle genes upon drug washout. A senescence‐associated secretory phenotype including various potentially immunogenic components was irreversibly induced. Phosphorylated CDK4 was detected in 80% of 47 MPMs indicating their sensitivity to CDK4/6 inhibitors. Its absence in some highly proliferative MPMs was linked to very high p16 (*CDKN2A*) expression, which was also observed in public datasets in tumours from short‐survival patients. Our study supports the evaluation of CDK4/6 inhibitors for MPM treatment, in monotherapy or combination therapy.

## Graphical abstract

CDK4/6 inhibition induces a deep proliferation arrest along with senescence in most pleural mesothelioma cell lines. In some cells and tumours, insensitivity is predicted by the absence of active T172‐phosphorylated CDK4, mostly due to elevated p16/*CDKN2A* levels consequent to defects or cyclin E‐dependent inactivation of pRb. Phospho‐CDK4 is nevertheless detected in most mesotheliomas, which should be sensitive to CDK4/6 inhibitors.

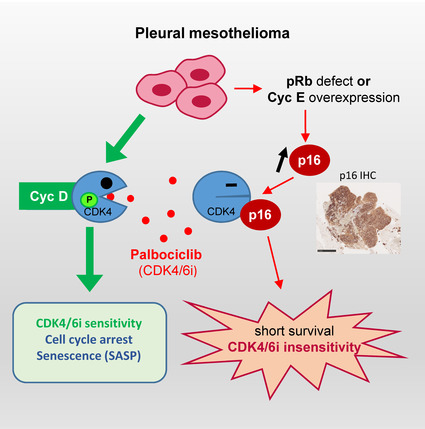

**Abbreviations**
2D‐gel: two‐dimensional gel
BrdU: 5‐bromo‐2'‐deoxyuridine
CDK: cyclin‐dependent kinase
CDK4/6i: inhibitors of CDK4 and CDK6
CP20M: counts per 20 million
DMSO: dimethyl sulfoxide
EdU: 5‐ethynyl‐2'‐deoxyuridine
EGFR: epidermal growth factor receptor
EMT: epithelial–mesenchymal transition
FBS: foetal bovine serum
FFPE: formalin‐fixed paraffin‐embedded
IGV: Integrative Genomics Viewer
IHC: immuno‐histochemistry
INK4: INhibitor of CDK4
MEK: MAP/ERK kinase
MPM: malignant pleural mesothelioma
MTT: 3‐[4,5‐dimethylthiazol‐2‐yl]‐2,5 diphenyl tetrazolium bromide
mTOR: mammalian target of rapamycin
PARP: poly(ADP‐ribose) polymerase
PBS: phosphate buffer saline
PI3K: Phosphoinositide 3‐kinase
pRb: retinoblastoma protein
qRT‐PCR: quantitative Reverse Transcription PCR
RNA‐seq: RNA‐sequencing
SA‐β‐gal: senescence‐associated beta‐galactosidase
SASP: senescence‐associated secretory phenotype
SDS/PAGE: sodium dodecylsulfate polyacrylamide gel electrophoresis
SNP: single‐nucleotide polymorphism
SRB: Sulforhodamine B
SV40: simian virus 40

## 1 Introduction

Malignant pleural mesothelioma (MPM) is a rare and aggressive tumour mostly associated with asbestos exposure. Despite the ban of asbestos in several countries, it remains a major public health concern worldwide. MPMs are classified into three main histological subtypes with different prognosis: epithelioid, biphasic and sarcomatoid [1]. As current treatment options (cisplatin + pemetrexed combined with bevacizumab if available) [2, 3, 4] only extend survival for a few months, alternative therapies with predictive markers based on the MPM biology are desirable [5, 6, 7, 8, 9, 10]. Recently, dual immune checkpoint inhibition with nivolumab plus ipilimumab has demonstrated superiority to platinum plus pemetrexed in MPMs with nonepithelioid histologies [11], but this remains questioned for epithelioid MPMs [2].

Malignant pleural mesotheliomas are mostly characterized by frequent deletions (50–80%) of the *CDKN2A/B* locus (encoding the CDK4/6 inhibitors p16^INK4A^ and p15^INK4B^) [8, 12, 13, 14], which are associated with shorter overall survival [15]. Other recurrent defects include mutations affecting the neurofibromatosis 2 (*NF2*) tumour suppressor encoding merlin and the BRCA1‐associated protein 1 (*BAP1*). NF2, as a key player in the Hippo pathway involved in cell contact growth inhibition [14], negatively regulates the expression of cyclin D1 [16]. These different defects are expected to deregulate the activity of CDK4 and cause the observed hyper‐proliferation. On the other hand, the onco‐suppressor pRb (*RB1*), the main target of CDK4, has been found to be intact in most MPMs, pointing out these tumours as paradigmatic targets for CDK4/6 inhibitors (CDK4/6i) [8, 12]. However, a recent study reported *RB1* deletions (possibly mostly monoallelic) in 26% of 118 MPM samples including in one‐third of tumours with *CDKN2A* deletions [17].

Pharmacological inhibition of CDK4/6 has emerged as a promising anti‐cancer strategy. Palbociclib, ribociclib and abemaciclib, combined with endocrine therapy, recently became a standard‐of‐care as first‐line treatments for advanced oestrogen receptor‐positive breast cancers. Despite mild side effects (cytopenia), these CDK4/6i induce an ‘unprecedented improvement of progression‐free survival’ [18] and also significantly improve the overall survival [19, 20]. In different cancer models, specific inhibition of CDK4/6 not only induces the cell cycle arrest but also a senescent‐like state [21, 22, 23, 24, 25] including a senescence‐associated secretory phenotype (SASP) [21, 26]. However, because CDK4/6 inhibition is often insufficient to fully control the tumour progression, rationally developed doublet or triplet combination therapies of CDK4/6i are evaluated in various cancers, for instance with kinase inhibitors (e.g. MEK, PI3K, mTOR, EGFR inhibitors, …) [21], genotoxic therapies [27, 28] and immunotherapies [21, 27, 29, 30].

Cyclin D‐CDK4/6 are the first CDK complexes to be activated during the G1 phase in response to mitogenic/oncogenic stimuli [31, 32, 33]. Their main function consists in the inactivation of the pRb (*RB1*) anti‐oncogene [34, 35]. Insensitivity to CDK4/6i is generally ascribed to the functional loss or inactivation of pRb [36]. However, CDK4 and CDK6 might also control the cell cycle progression by phosphorylating a growing list of other proteins including p107, p130, FoxM1 and Smad3 [32, 35, 37, 38, 39, 40, 41]. pRb inactivating phosphorylations in committed cells are maintained by a positive feedback loop linking pRb to E2F‐dependent transcription of *CCNE1* (cyclin E1), which activates CDK2, further enhancing the inactivation of pRb [42]. CDK4 activity requires its binding to a cyclin D (*CCND1‐3* genes) competed by INK4 CDK4 inhibitors like p16 (*CDKN2A‐D* genes) [33, 43]. To be active, CDK4 also needs to be phosphorylated on T172 [44]. Our group has identified the activating T172‐phosphorylation of cyclin D‐bound CDK4 as the last distinctly regulated step in CDK4 activation, determining the cell cycle commitment in pRb‐proficient cells [31, 45, 46, 47, 48, 49, 50]. In breast cancer tumours and cell lines, we have reported that the highly variable abundance of phospho‐CDK4 signals the presence or absence of active CDK4 targeted by CDK4/6i, which was associated with the sensitivity or insensitivity of tumour cells to palbociclib [51]. CDK4 T172‐phosphorylation might thus be the best biomarker of potential tumour sensitivity to CDK4/6i.

Discordant results were reported in the few studies evaluating CDK4/6i in MPM cell lines [17, 52, 53]. Nevertheless, a single‐arm, open‐label, phase II trial of 27 MPM patients recently showed a promising clinical activity of abemaciclib [54]. Here, we demonstrate and characterize the unique responsiveness of most MPM cell lines to CDK4/6 inhibition. In a few cell lines, a complete insensitivity to palbociclib is nevertheless observed. It is mostly associated with the absence of phosphorylated CDK4, which can occur even in the absence of *RB1* defect. CDK4 phosphorylation is detected in about 80% of MPM tumours, which are therefore predicted to be responsive to CDK4/6i. CDK4 phosphorylation is nevertheless undetectable in a subset of MPMs characterized by especially high p16/*CDKN2A* expression. The occurrence of this subset of high *CDKN2A* expressers is confirmed in publicly available datasets in patients with short survival. It might have to be considered in future clinical settings involving CDK4/6i.

## 2 Methods

The antibodies and drugs used in this work are listed in Table S1.

### 2.1 Cell culture

MeT‐5A, NCI‐H2052, NCI‐H2452, NCI‐H28 and MSTO‐211H were obtained from ATCC (Manassas, VA, USA), MPP89 from Interlab Cell Line Collection (Genoa, Italy), DM‐3 and RS‐5 from DSMZ (Brunswick, Germany), and NCI‐H2369 from Wellcome Sanger Institute. These authenticated cell lines were passed for fewer than 4 months after receipt. Meso11, Meso13, Meso34 and Meso56 were established in the laboratory of Marc Gregoire and Christophe Blanquart (CRCI2NA, Nantes) from pleural effusions from patients who had not received any chemotherapy [55]. They were characterized phenotypically for genetic alterations in key genes of mesothelial carcinogenesis including *CDKN2A*, *CDKN2B*, *BAP1*, *NF2*, *LATS2* and *TP53* using a targeted sequencing described in the study cited herein [56] and karyotyping (GSE134349) [57]. MPM_04 to MPM_66 cell lines are primary mesothelioma cell lines obtained from surgical resections, pleural biopsies or malignant pleural fluids of confirmed MPM cases in the research teams of Marie‐Claude Jaurand and Didier Jean (Centre de Recherche des Cordeliers, Paris). They were previously characterized for genetic alterations using the same targeted sequencing [56] and some of them were characterized for copy number variations by single‐nucleotide polymorphism (SNP) arrays. The patient‐derived cell lines were used in several studies showing their relevance to MPM primary tumours [5, 55, 58, 59, 60]. These cell lines were authenticated based on specific gene mutations. DM‐3 and RS‐5 cells were cultured in NCTC‐109 medium (Gibco, Carlsbad, CA, USA) supplemented with antibiotics, sodium pyruvate (1 mm), glutamine (2 mm), N‐Acetyl‐L‐cysteine (1 mm, Sigma Aldrich, St Louis, MO, USA) and 10% (for RS‐5) or 20% (for DM‐3) FBS (Gibco). All the other cells were cultured in RPMI medium (Gibco) supplemented with antibiotics, sodium pyruvate (1 mm) and 10% FBS.

### 2.2 Human MPM samples

Fresh frozen mesothelioma and normal pleura samples were obtained from Biobanque of Erasme Hospital (ULB), French MESOBANK (Centre Léon Bérard, Lyon) or UZA Tumor bank (Antwerp University Hospital, Belgium) with a signed informed consent for each subject. For a prospective study, MPMs were resected at the Thoracic Surgery Department of Hôpital Erasme Hospital (*n* = 4). This study was performed in accordance with the Declaration of Helsinki and approved by the Ethics Committees of Jules Bordet Institute (n^r^ 2288), Erasme academic hospital (P2014/312/B079201421880) and UZ Antwerpen (ref. 16/9/98). Tissue samples from French MESOBANK were collected in agreement with all applicable laws, rules, and requests of French and European government authorities. These samples were prepared by BB‐0033‐00050, CRB Centre Léon Bérard, Lyon France.

### 2.3 DNA synthesis

DNA‐replicating cells were identified from duplicated dishes by incubation with bromodeoxyuridine (BrdU) for 1 h and counted at the microscope as described [61]. For the slow‐growing RS‐5 cells, BrdU incubation was done for the last 24 h of a 48‐h treatment with palbociclib.

Alternatively, cells were seeded in triplicates in 96‐well plates (10^4^ cells per well) and BrdU was replaced by EdU (5‐ethynyl‐2′‐desoxyuridine, Thermo Fischer Scientific, Waltham, MA, USA). EdU (10 μm final concentration) was incorporated 1 h before fixation and nuclei staining using 4% formalin (Sigma‐Aldrich) and 5 μg·mL^−1^ Hoechst 33342 (Thermo Fischer Scientific) solution for 15 min at room temperature. Permeabilization was achieved with 0.3% Triton X‐100 (Sigma‐Aldrich) solution for 20 min at room temperature. Cells were rinsed with 1% Bovine Albumin Fraction V (Thermo Fischer Scientific) solution. Staining was done with 1 mm Copper (II) sulphate solution (Sigma‐Aldrich), 100 mm L‐Ascorbic acid (Sigma‐Aldrich) and 3 μm Alexa Fluor™ 647 Azide (Thermo Fischer Scientific) for 30 min at room temperature. Cells were rinsed with 1% Bovine Albumin Fraction V (Thermo Fischer Scientific) solution. Nuclei counting and Edu positive cells were assessed with an Operetta High Content Imaging System (PerkinElmer, Waltham, MA, USA).

Pictures of abnormal nuclei were acquired using an epifluorescence microscope Zeiss Axioplan 2 (Zeiss, Jena, Germany) equipped with a Spot RT3 camera and the spot 5.2 imaging software (Spot Imaging, Sterling Heights, MI, USA).

### 2.4 Cell growth assays

Cells were seeded in triplicates in 96‐well plates and treated the day after with serial dilutions of CDK4/6i (palbociclib, ribociclib or abemaciclib) for 48 h (MTT assay) or 144 h [Sulforhodamine B (SRB) assay]. MTT and SRB assays were performed as described in the study cited herein [51].

### 2.5 Clonogenic assays

5 × 10^3^ cells were seeded in 6‐well plates and treated the day after with the indicated drugs for 10 days. For drug washout, cells were washed twice with phosphate‐buffered saline (PBS) before incubation in complete medium for 7 days. After removal of culture medium, cells were washed with PBS and fixed in a 10% formalin solution (Sigma‐Aldrich) for 10 min. Cells were washed again with PBS and stained for 30 min with 0.05 % crystal violet solution (Sigma‐Aldrich), washed thoroughly, and air‐dried. Photographed pictures of the plates were quantified with the ‘ColonyArea’ plugin [62] in imagej software.

### 2.6 SA‐β‐gal activity assay

5 × 10^3^ cells (or 10^3^ for the control) were seeded in 6‐well plates and treated the day after with DMSO or 1 μm palbociclib for 9 days. SA‐β‐gal activity was measured using the SA‐β‐gal staining kit (Cell Signaling Technology, Danvers, MA, USA) according to the manufacturer's protocol. After overnight incubation at 37 °C without CO_2_, the percentage of SA‐β‐gal positive cells was determined by counting at least 500 cells per well.

### 2.7 Cytokine array

3 × 10^5^ to 10^6^ cells were plated in 9‐cm Petri dishes and treated with DMSO or 1 μm palbociclib for 9–10 days. Cells were trypsinized when necessary and medium was changed every 3–4 days. Culture supernatants were collected 48 or 72 h after the last medium replacement and cells were lysed to perform immunoblotting and allow normalization of media based on protein quantification. Supernatants were sent to Tebu‐Bio (Le Perray en Yvelines, France) for analysis on a custom Quantibody Multiplex Elisa Array (RayBiotech, Peachtree Corners, GA, USA).

### 2.8 Immunoprecipitations and pRb‐kinase assay

Co‐immunoprecipitations were performed as described [47, 63]. pRb‐kinase activity of immunoprecipitated CDK complexes was measured by *in vitro* incubation with ATP and a fragment of pRb, as described [63, 64].

### 2.9 Western blots

Equal amounts of whole cell extract proteins or immunoprecipitates were separated by SDS/PAGE and immunodetected.

For 2D‐gel electrophoresis, cells were lysed in a buffer containing 7 m urea and 2 m thiourea. Frozen tumour slides (minimum seven sections of 7 μm per sample) or frozen tissue powder obtained by cryogrinding were solubilized as described [51]. Proteins were separated by isoelectric focusing on immobilized linear pH gradient strips pH 5–8 for CDK4 (BioRad, Hercules, CA, USA) or pH 3–10 for CDK2 (Amersham Biosciences, GE Healthcare Europe, Diegem, Belgium) before separation by SDS/PAGE [47, 65].

Chemiluminescence detections were captured on films or with a Vilber‐Lourmat Solo7S camera and quantified using the Bio1D software (Vilber‐Lourmat, Marne‐la‐Vallée, France). The profile of CDK4 separated by 2D‐gel electrophoresis has been characterized previously [47, 51]. The most basic form (spot 1) corresponds to unmodified CDK4. The most acidic form (spot 3) had been identified as T172‐phosphorylated CDK4 form using several approaches including a T172‐phosphospecific antibody. Another yet unidentified modified CDK4 form (spot 2) does not incorporate [32P] phosphate. The background‐subtracted volume ratio (spot 3/spot 2) was used to define the type of CDK4 modification profile of the tumours [51]. A profile A (Absent) was attributed to the sample when its ratio was below 0.025. A profile L (Low) was attributed to the sample if this ratio was between 0.025 and 0.5, while a profile H (High) was given for ratio above 0.5.

### 2.10 γH2AX immunofluorescent labelling

Cells were fixed with 2% paraformaldehyde for 90 s followed by cold methanol for 10 min and permeabilized with 0.1% Triton X‐100 for 10 min. After a blocking step in 3% PBS/BSA with normal sheep serum (1/20), cells were incubated overnight at 4 °C with the anti‐phospho‐histone H2AX (Ser139) antibody (1/400). Cells were washed three times with PBS/BSA before incubation for 2 h with Alexa Fluor 488 anti‐rabbit (1/500) at room temperature. Cells were washed once with PBS/BSA and once with PBS, and counterstained with propidium iodide (0.5 μg·mL^−1^). After washing with water, coverslips were mounted with Prolong Glass Antifade (Invitrogen). Images were acquired using an epifluorescence Zeiss Axioimager Z1 microscope equipped with Zeiss AxioCam MRc5 (Zeiss). γH2AX staining was evaluated in minimum 100 cells per condition.

### 2.11 Immunohistochemistry

Immunohistochemical stainings of Ki‐67 and p16 were performed using standard routine protocols and scored by a pathologist. Pictures were acquired with a Moticam Pro camera connected to Motic AE31 microscope or a NanoZoomer digital scanner (Hamamatsu Photonics, Hamamatsu, Japan) at 40× resolution.

### 2.12 RNA‐sequencing

Total RNA was isolated from cell lines or frozen tumour tissues using the RNeasy Mini Kit according to the manufacturer's protocol (Qiagen, Hilden, Germany). RNA yield and purity were assessed using a Fragment Analyzer 5200 (Agilent Technologies, Massy, France). 100 ng of RNA was used to create indexed cDNA libraries using the NEBNext Ultra II Directional RNA Library Prep Kit for Illumina (New Englands Biolabs, Ipswich, MA, USA) following manufacturer's protocol. The multiplexed libraries were loaded on a NovaSeq 6000 (Illumina, San Diego, CA, USA) using a S2 flow cell and sequences were produced using a 200 Cycles Kit. Paired‐end reads were mapped against the human reference genome GRCh38 using star software to generate read alignments for each sample. Annotations Homo_sapiens.GRCh38.90.gtf were obtained from ftp.Ensembl.org. After transcripts assembling, gene level counts were calculated with HTSeq and normalized to library size to obtain counts per 20 million reads (CP20M).

### 2.13 Analysis of RNA‐sequencing data

The CP20M count matrix was filtered to keep genes expressing a minimum of 100 counts in at least one sample. Differentially expressed genes with a fold change (FC) > 1.5 or < 1/1.5 and false discovery rate (FDR) < 0.05 were identified using DESeq2 in r or in the iDEP.91 platform. The model ‘treatment + paired’ was used to allow pairwise comparison between the treated and control samples within each experiment. For pathway analysis, the ranked gene list was used in Gene Set Enrichment Analysis (gsea) Preranked using the ‘Hallmarks’ and ‘Kegg’ Molecular Signatures database (MSigDB) gene sets v7.2. Fridman_senescence_up (from the ‘Curated’ gene sets), gene sets containing epithelial and mesenchymal markers previously identified in MPM [59] and the gene sets up‐ and down‐regulated as part of the immune resistance programme described by Jerby‐Arnon et al. [66] were also used. Heatmaps were generated using the heatmap.2 package in r. A proliferation score was calculated by using the median expression of the genes of the Cell Cycle Progression (CCP) signature described in the study cited herein [67].

### 2.14 Viral genome detection and quantification

The presence of viral genome in MPM tumours and cell lines was explored by aligning the raw reads using the star algorithm to a custom GTF file built with the genomic sequences of viruses. This GTF file was built by downloading the fasta files sequences of the viral genomes from the NCBI database and converting them into a GTF file using a dedicated script in r (available upon request). Each viral genome is recorded as a single gene in this GTF file. Gene level counts were normalized to library size to obtain counts per 20 million reads (CP20M). A threshold of minimum 20 counts was applied to minimize the rate of false‐positive hits in virus detection.

### 2.15 Analysis of splicing junctions

The genomic coordinates of the *RB1* gene exons were first extracted from Ensembl with a r script extracting data corresponding to the ENSG00000139687 id using the library biomaRt. The coordinates from the introns are computed from the coordinates of the exons. The genomic position at −200 nucleotide of the transcription start site is used as the starting coordinate of the upstream sequence. The length of the exons was computed based on the coordinates of the exons. Next, for a given exon, we identify the most distant exons in 5′ for which the sequence could be included after splicing in a read of 97 nucleotides ending in the given exon. The *RB1* gene exon genomic coordinates are converted to a genomic Range object using the makeGRangesFromDataFrame function of the GenomicRanges library. BAM files are opened using the BamFile function of the Rsamtools library. The function scanBam of the GenomicRanges library is used to extract the reads, their position and the cigars information. For each read falling in a genomic coordinate range corresponding to an exon for which at least 10 reads were recorded, the script identifies whether the coordinate of the start of the read is located within the expected exons located upstream of the considered exon defined above. The number of reads for which the start coordinates are within an expected exon defined for each exon and each sample is divided by the number of informative reads to compute the expected splicing ratio. Commented scripts to reproduce the data are available upon request.

A heatmap of these proportions was drawn with the heatmap.3 function from the heatmap3 package. If the number of reads for a given exon is below 10, the data are considered as not informative and appear as a black cell in the heatmap.

### 2.16 Quantitative Real Time ‐PCR (qRT‐PCR) analysis

Total RNA (1.5 μg) was reverse transcribed in a final volume of 50 μL using the High Capacity cDNA Reverse Transcription kit (Thermo Fisher Scientific, Waltham, MA, USA). qRT‐PCR reactions were performed using TaqMan probes (Thermofisher) and the high throughput BioMark HD system (Fluidigm) following manufacturer's instructions. Expression data (*C*_t_ values) were acquired using the Fluidigm Real Time PCR Analysis software. The mean of the following five housekeeping genes *ACTB* (Hs01060665_g1), *CLTC* (Hs00964504_m1), *GAPDH* (Hs02758991_g1), *TBP* (Hs00427620_m1) and *RNA18S* (Hs03928990_g1) was used for the normalization of CDKN2A (Hs00923894_m1) expression data (−Δ*C*_t_).

### 2.17 Single‐nucleotide polymorphism arrays

Some MPM cell lines were characterized for copy number variations using Illumina HumanOmniExpress‐24 v1.0 BeadChip SNP arrays. Integragen SA (Evry, France) carried out hybridization, according to the manufacturer's recommendations. The beadstudio software (Illumina) was used to normalize raw fluorescent signals and to obtain log R ratio (LRR) and B allele frequency (BAF) values. Asymmetry in BAF signals due to bias between the two dyes used in Illumina assays was corrected using the tQN normalization procedure [68]. We used the circular binary segmentation algorithm [69] to segment genomic profiles and assign corresponding smoothed values of log R ratio and B allele frequency. The Genome Alteration Print (GAP) method was used to determine the ploidy of each sample, the level of contamination with normal cells and the allele‐specific copy number of each segment [70].

### 2.18 Targeted DNA‐sequencing

Genomic DNA was extracted from frozen tissue using the QiaAmp Mini Kit or the DNA/RNA extraction mini kit from Qiagen. DNA was quantified and quality checked with the Qubit fluorimeter or the Quant‐iT PicoGreen dsDNA Assay Kit (Thermo Fisher Scientific). Massive parallel sequencing was performed using targeted‐capturing of the 165 genes included in the ‘Solid and Haematological tumours’ panel (BRIGHTCore, Brussels, Belgium). 150 ng of genomic DNA was fragmented and processed to construct libraries with barcodes, which were hybridized with the DNA panel. The libraries were sequenced on Illumina NovaSeq 6000 with a coverage of 1500×.

### 2.19 DNA‐sequencing analyses

Copy number calling. We derived genome‐wide copy number profiles using the off‐target reads from our targeted panel, as these provide a uniform shallow‐coverage whole‐genome sequencing experiment. We first remove the on‐target regions from the bed definition of the target with padding of 1000 bp upstream and downstream. Then we bin the rest of the genome in 30 kb bins and count the number of reads with MAPQ > 30 falling in each bin to obtain a read‐count track genome‐wide. We divide the number of reads in each bin by the average number to get a ratio *r*, which can be expressed as a function of the purity ρ, the average number of copies (or average ploidy ψ) and the local number of DNA copies nT: *r* = (n_T ρ + 2(1−ρ))/ψ. We take the log of this track and segment it with circular binary segmentation [71] from the r package DNAcopy v1.64.0 with default parameters. Then for each value of the ploidy ψ∈[1.5,5] by 0.01 and purity ρ∈[.2,1] by 0.01 we fit n_T of each segment from the first equation n_T = (ψr−2(1−ρ))/ρ. We calculate the sum of Euclidean distances between n_T and the closest integer values, that is, round (n_T), and then select the combination of values of ψ and ρ that minimizes this distance. For copy number of exons from on‐target reads, we counted the number of aligned reads with MAPQ > 30 at the centre of each exon target and divided the counts by the counts from the identified diploid sample L2 to normalize for different target‐capture efficiency.

RNA splice junctions. From the gene annotation from Ensembl v104.38, we looked at the mate coordinates of reads aligning at the exon junctions, showing abnormal amounts of noncanonical junctions in *RB1* of four of the samples.

### 2.20 Analysis of mutations

Integrative Genomics Viewer software (igv version 2.7.2, Broad Institute, Cambridge, MA, USA) was used to visualize mutations from RNA‐seq and targeted DNA‐sequencing.

### 2.21 Analysis of publicly available RNA‐seq data

RNA‐seq data were retrieved from The Cancer Genome Atlas (TCGA‐Meso, *n* = 86) and from Genentech/gRED (*n* = 211) [12]. Raw counts were normalized to library size to obtain counts per 20 million reads (CP20M). RNA‐seq data were linked to mutation and copy number alteration (CNA) data to identify samples with *RB1* alterations. BAM files were visualized in igv to validate the *RB1* status. Overall survival was compared between patients expressing high, intermediate or low *CDKN2A*; or high or low *CCNE1*. For *CDKN2A*, low expression was < 100, intermediate expression was from 100 to 1000 and high expression was > 1000. For *CCNE1*, the stratification threshold between high and low expression was set at 200 (which corresponds approximately to the mean + 1 SD). For *RB1* expression, the threshold was set at 400 (which corresponds approximately to the mean − 1.5 SD). Of note, different stratification thresholds were used for our own data based on the different distribution of the data. For *CDKN2A*, a high expression was considered from 600; for *CCNE1*, high expression was considered from 100 and for *RB1*, low expression was considered below 900.

### 2.22 Statistical analysis

Statistical analysis was performed using graphpad prism 6 (GraphPad Software, La Jolla, CA, USA) except for the Fisher's exact test, which was performed using BiostaTGV (https://biostatgv.sentiweb.fr). The two‐sided unpaired Student's *t*‐test was used for comparison between two groups. Multiple group comparisons were done using one‐way ANOVA followed by Holm–Sidak's multiple comparison tests or the Kruskal–Wallis test followed by Dunn's multiple comparison tests, as stated in the figure legends. Correlation was evaluated with a Spearman's rank test. Comparison of Kaplan–Meier survival curves was performed using the Mantel–Cox log‐rank test. The Fisher's exact test (two‐sided) was used for the analysis of contingency tables.

## 3 Results

### 3.1 Most MPM cell lines exhibit a high sensitivity to CDK4/6 inhibition, which correlates with the presence of phosphorylated CDK4

**Fig. 1:**
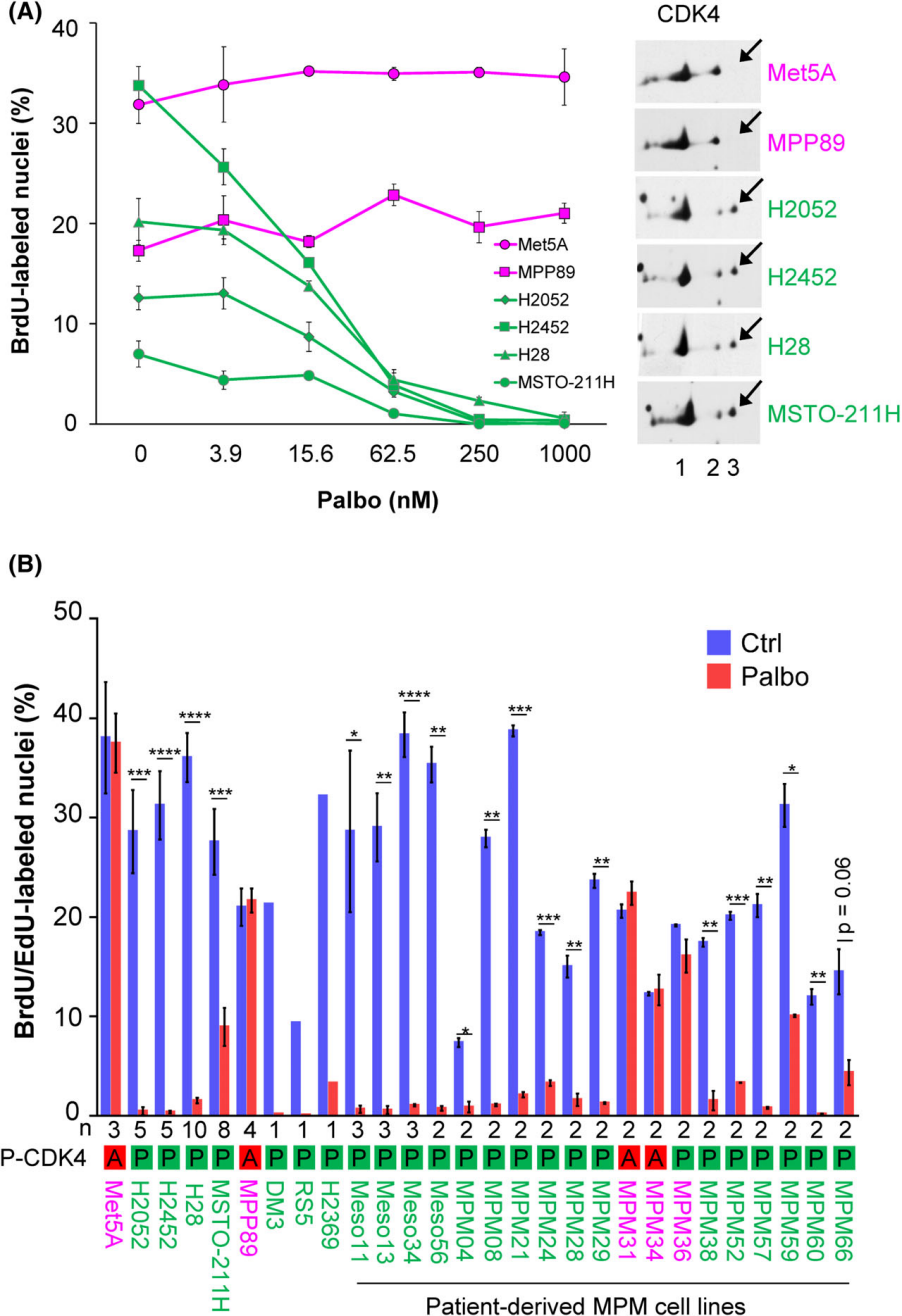
Sensitivity of malignant pleural mesothelioma (MPM) cell lines to palbociclib is associated with CDK4 phosphorylation. Sensitive cell lines are in green, resistant cell lines are in magenta. (A) Left panel. DNA synthesis in MPM cell lines treated for 24 h with increasing concentrations of palbociclib (Palbo). Data show one representative experiment (mean ± SD of duplicated dishes). Right panel. CDK4 immunodetection after separation by 2D‐gel electrophoresis. Arrows indicate the position of the T172‐phosphorylated form of CDK4 (spot 3). (B) DNA synthesis in cells treated for 24 h with DMSO (Ctrl) or palbociclib (Palbo) at 1 μm. Data represent mean ± SEM of *n* independent experiments, as mentioned below the graph. P‐CDK4 indicates the phosphorylation status of CDK4: A, absent and P, phosphorylated. *P < 0.05, **P < 0.01, ***P < 0.001, ****P < 0.0001 (Student's *t*‐test).

We selected a panel of well‐characterized MPM cell lines representative of the three histotypes and comprising both commercial and patient‐derived cell lines including low‐passage ones (Table S2). MPP89 was chosen because it harbours a mutated *RB1*. Mesothelial cells immortalized by SV40 (MeT‐5A) [72, 73] were also used as a model of pRb‐deficient cells. Treatment with palbociclib for 24 h prevented the cell cycle progression in a concentration‐dependent manner in 23 of 28 cell lines (Fig. 1A, Fig. S1). In the majority of these, 1 μm palbociclib reproducibly reduced the number of DNA replicating cells by more than 90% (Fig. 1A,B). The effect of CDK4/6 inhibition was less pronounced when measured using MTT or sulforhodamine viability assays, confirming the cytostatic effect of palbociclib (Fig. S1). Similar results were obtained with ribociclib and abemaciclib (Fig. S1A,B).

In parallel, we evaluated in these cell lines the presence of the activated form of CDK4 phosphorylated on T172. This was achieved by 2D‐gel electrophoresis separation of CDK4 forms as previously characterized [47, 51] (Fig. 1A, Fig. S1). Whereas the T172‐phosphorylated form of CDK4 was detected in all the palbociclib‐sensitive cell lines, it was absent in four of five resistant ones (MPP89, MeT‐5A, MPM_31, MPM_34; Fig. 1B, Fig. S1). With the noticeable exception of resistant MPM_36 cells that displayed a low level of CDK4 phosphorylation (Fig. S1), the presence or absence of CDK4 T172‐phosphorylation thus correctly predicted the sensitivity or insensitivity to CDK4/6 inhibition in MPM cell lines, similarly to our initial observations in breast cancer cell lines [51].

### 3.2 Absence of CDK4 phosphorylation in resistant cells is due to high p16 levels associated or not with pRb defect

**Fig. 2:**
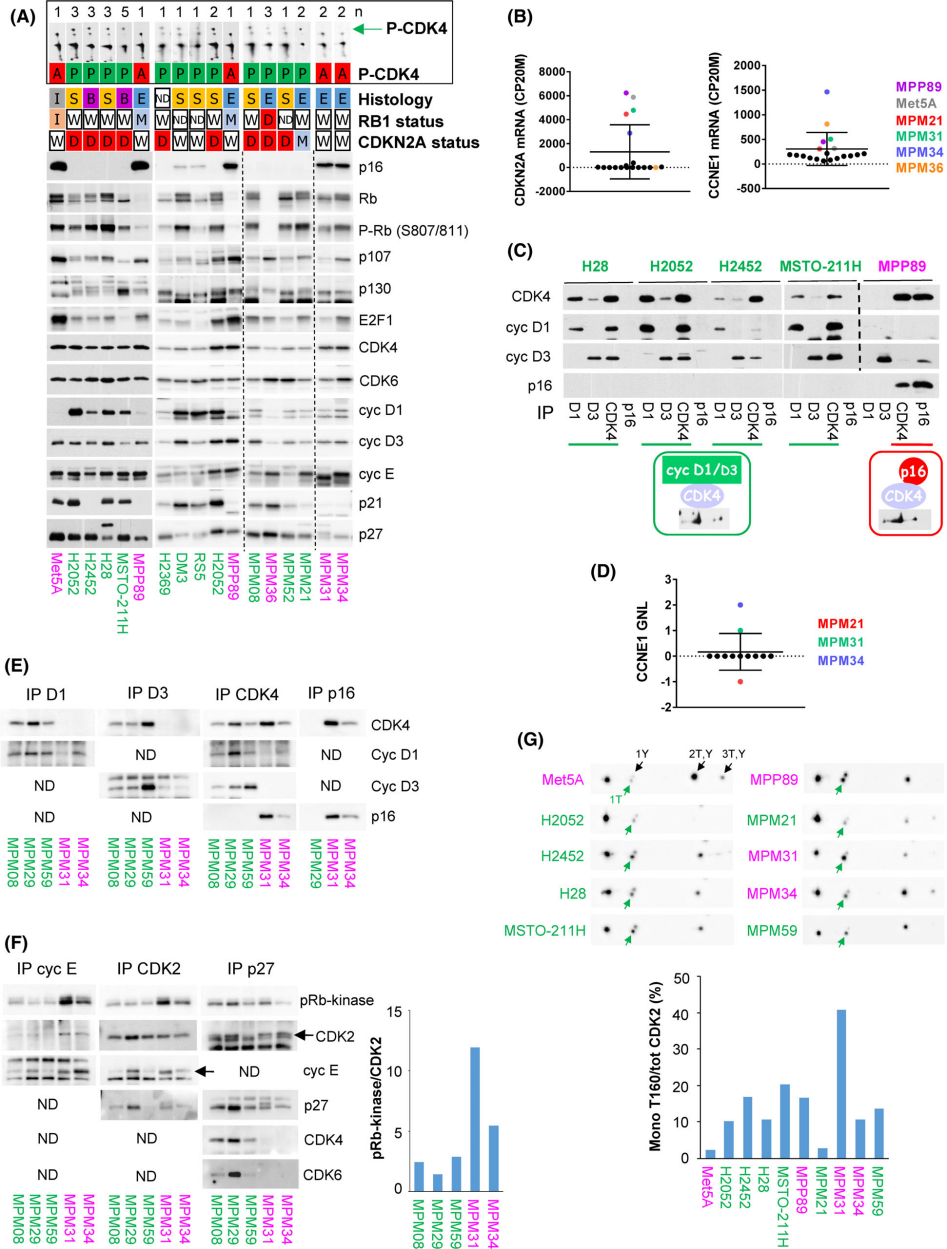
Absence of CDK4 phosphorylation in resistant cells is due to high p16 levels associated with pRb defect or high CDK2 activity. Sensitive cell lines are in green, resistant ones are in magenta. (A) Western blot analysis of the indicated proteins and profile of CDK4 separated by 2D‐gel electrophoresis (the green arrow indicates the position of the phosphorylated form of CDK4). Vertical dashed lines separate parts of the same blot that were assembled. H2052 and MPP89 were loaded on each gel to compare protein expression between the different gels. When the 2D‐gel electrophoresis was performed several times (*n* as indicated at the top of the panel), one representative image is shown. The following data are given for each cell line: CDK4 phosphorylation profile (P‐CDK4, A: absent, P: phosphorylated), histological subtype (E: epithelioid, S: sarcomatoid, B: biphasic, I: immortalized mesothelial cells, ND: not determined), status of *RB1* and *CDKN2A* genomic locus (W: wild‐type, I: wild‐type but inactivated by SV40, D: deleted, M: mutated, ND: not determined). (B) *CDKN2A* and *CCNE1* mRNA expression extracted from RNA‐seq data and normalized relative to the library size in counts per 20 million reads (CP20M). *N* = 19 cell lines. Data represent mean ± SD. The mean of two independent experiments (*n* = 2) was used except for MPM_36 and MPM_66 (*n* = 1); and for H2452, H28, MSTO‐211H and MPM_59 (*n* = 3). (C) Immunodetection of the indicated proteins from CDK4 complexes co‐immunoprecipitated (IP) using anti‐cyclin D1, cyclin D3, CDK4 or p16 antibodies. *N* = 1. (D) Gain/normal/loss (GNL) score of *CCNE1* locus analysed by single‐nucleotide polymorphism array. *N* = 12 cell lines. Data represent mean ± SD. (E) Immunodetection of the indicated proteins from CDK4 complexes co‐immunoprecipitated (IP) using anti‐cyclin D1, cyclin D3, CDK4 or p16 antibodies. ND: not done (less relevant). *N* = 1. (F) Co‐immunoprecipitation (IP) of CDK2 complexes using anti‐cyclin E1, CDK2 or p27 antibodies followed by pRb‐kinase assay and immunodetection of the indicated proteins. ND: not done (less relevant). Quantification of phospho‐pRb signal normalized to CDK2 is shown on the right. *N* = 1. (G) CDK2 immunodetection after separation by 2D‐gel electrophoresis. Arrows indicate the various phosphorylated forms of CDK2. 1T (active CDK2 in green): mono‐T160 phosphorylation, 1Y: mono‐Y15 phosphorylation, 2T,Y: double phosphorylation on T160 and Y15 and 3T,Y: triple phosphorylation on T160, Y15 and T14. Quantification of the proportion of active CDK2 phosphorylated only at T160 (mono‐T160) over total CDK2 is displayed below the detections. *N* = 1.

To better understand the mechanisms involved in palbociclib resistance and in the lack of CDK4 phosphorylation, the cell lines were characterized by immunodetection of key cell cycle‐related proteins and by RNA‐sequencing (RNA‐seq) for 19 of them. Insensitive cell lines lacking phospho‐CDK4 were distinguished by strongly elevated p16 levels, consistent with high *CDKN2A* mRNA expressions (Fig. 2A,B; Fig. S2; Table S2). In that situation, co‐immunoprecipitation experiments showed that p16 prevents the activating phosphorylation of CDK4 by impairing its binding to D‐type cyclins (Fig. 2C). Constitutive loss of function of pRb, the most obvious driver of resistance to CDK4/6i, is known to lead to elevated *CDKN2A* mRNA levels [74, 75]. Inactivation of pRb by SV40 in MeT‐5A and its mutation in MPP89 could thus explain the high levels of p16 in these two cell lines and the resulting impairment of CDK4 phosphorylation. This role of p16 was further demonstrated by the specific case of MPM_36 cells, which harbours both *RB1* and *CDKN2A* biallelic deletions (Fig. 2A, Table S2). Indeed, MPM_36 was the only pRb‐deficient palbociclib‐resistant cell line that presented a detectable phosphorylation of CDK4; reproducing the exceptional situation observed in DU4475 breast cancer cells [51].

In contrast, MPM_31 and MPM_34 presented high *CDKN2A* mRNA and p16 levels despite normal expression and phosphorylation of pRb (Fig. 2A,B, Fig. S2). No *RB1* defect (mutation or deletion) was found by RNA‐seq covering all *RB1* exons of the MPM_31 and MPM_34 resistant cells (Table S2). Inactivation of pRb by SV40 large T [76] was also excluded by RNA‐seq analysis (Table S3). On the other hand, these cells were among those expressing the highest abundances of cyclin E1 at the protein and mRNA levels (Fig. 2A,B, Fig. S2A), consistent with the amplification of the *CCNE1* gene locus detected by SNP array (Fig. 2D). In addition, MPM_31 displayed an abnormal electrophoretic migration of p27 (two bands; Fig. 2A, Fig. S2A), which we found to be associated with a I119T mutation of one *CDKN1B* allele that was previously reported in other cancers (Table S2) [77, 78, 79]. We also observed an abnormal electrophoretic migration of cyclin E1 in MPM_31 (Fig. 2A, Fig. S2A), which most likely indicated a post‐translational modification because it could not be explained by any genetic alteration detected by RNA‐seq of *CCNE1*. To evaluate the effect of these peculiarities, we analysed by co‐immunoprecipitation the formation and activity of different CDK complexes in MPM_31 and MPM_34 as compared to three palbociclib‐sensitive primary MPM cell lines (Fig. 2E,F). Similarly to MPP89 and MeT‐5A, CDK4 was bound to p16 but neither to cyclin D1 nor cyclin D3 in MPM_31 and MPM_34 (Fig. 2E). A much higher pRb‐kinase activity was associated with CDK2 and cyclin E1 in these two resistant cell lines (Fig. 2F). Interestingly, an increased pRb‐kinase activity was also associated with p27 in MPM_31 cells. This might be ascribed to p27‐bound CDK2 complexes because no association of p27 with CDK4 or CDK6 was detected in this cell line (Fig. 2F), consistent with the main association of CDK4 with p16 (Fig. 2E). As analysed by 2D‐gel electrophoresis of CDK2 [65], the relative proportion of the active form of CDK2 phosphorylated on T160, but not on Y15 or T14, was also more elevated in MPM_31 cells (Fig. 2G), suggesting a particularly elevated cdc25 phosphatase activity. Whereas high expression of *CCNE1* is a well‐known and general mechanism of resistance to CDK4/6i [36, 80, 81, 82], these different observations suggest (for the first time to the best of our knowledge) that a constitutive overactivation of cyclin E1‐CDK2 and the resulting inactivation of pRb by phosphorylation can also preclude the activation of CDK4, directly leading to insensitivity to CDK4/6i.

The detection of T172‐phosphorylated CDK4 was a more consistent biomarker of sensitivity to palbociclib than the expression of any other key cell cycle regulatory protein. Indeed, pRb was detected not only in all the palbociclib‐sensitive MPM cell lines but also in four of five resistant ones, though at much reduced levels in the *RB1*‐mutated MPP89 cells (Fig. 2A). The mRNA level of *CDK6* was more variable than *CDK4*, but in contrast with other studies [83, 84, 85], cells with high *CDK6* levels including H28 and MPM_57 did not show any resistance to palbociclib (at IC50 or residual proliferation, Table S2, Fig. S2C). The only exception was the insensitive MPM_36 that uniquely combines deletions of *RB1* and *CDKN2A* and high expression of *CCNE1* and *CDK6*. The *CDK4* mRNA level also did not correlate with the sensitivity to CDK4/6i (Fig. S2D). Cyclin D1 was less abundant in all the insensitive *RB1*‐deficient cells, but it was present in MPM_31 and MPM_34 cells. p16 was highly expressed in four of five insensitive cell lines, but it was also present at moderate levels in the DM‐3 and RS‐5 sensitive cell lines. By contrast, p16 was undetectable in 21 of 23 sensitive cell lines, mostly due to deletion of *CDKN2A* or its mutation (paradoxically associated with elevated *CDKN2A* RNA levels in MPM_21 cells; Fig. 2A,B; Table S2). In MPM_59, loss of p16 expression might be due to methylation of the *CDKN2A* locus or mutation in its promoter region, since its RNA levels were low while no deletion or mutation were detected. It should be noted that MPM cell lines expressing normal levels of wild‐type p16 grew very slowly, precluding any detailed evaluation. Two such patient‐derived cell lines had to be excluded from our study because of insufficient EdU incorporation.

In brief, our results show that the cell cycle resistance to palbociclib was associated not only to pRb defects but also to the absence of CDK4 phosphorylation due to high p16 levels, even in the presence of normal pRb.

### 3.3 Prolonged palbociclib treatment induces senescence in MPM cell lines

**Fig. 3:**
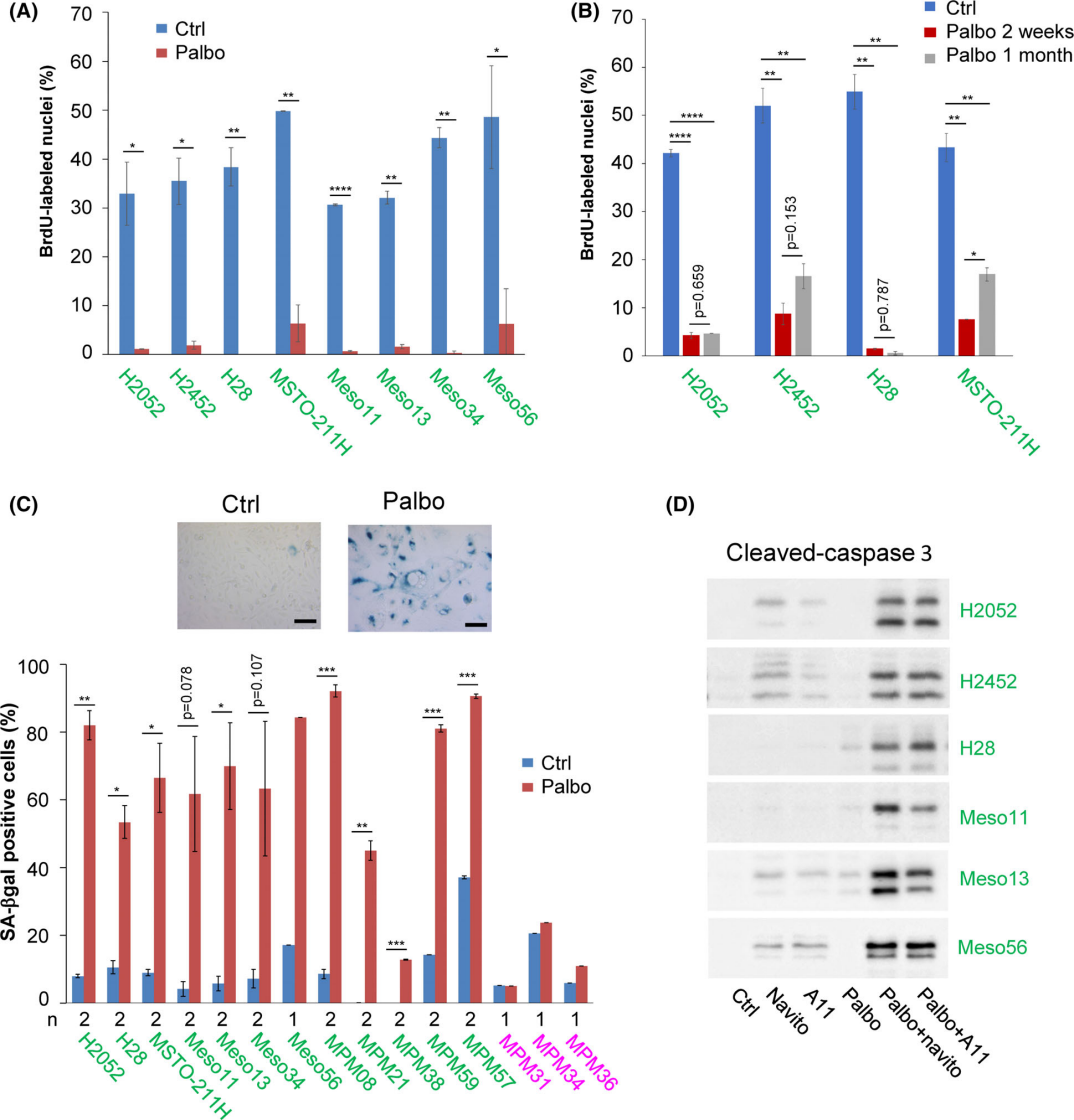
Prolonged palbociclib treatment induces senescence in MPM cell lines. Sensitive cell lines are in green, resistant ones are in magenta. (A) DNA synthesis in cells treated for 2 weeks with DMSO (Ctrl) or palbociclib (Palbo) at 1 μm. Data are presented as mean ± SEM of two independent experiments (*n* = 2). (B) DNA synthesis in cells treated for 2 weeks or 1 month with DMSO (Ctrl) or palbociclib (Palbo) at 1 μm. Data are presented as mean ± SEM of two independent experiments (*n* = 2). (C) Representative SA‐β‐gal staining of a sensitive cell line (H2052) treated for 9 days with DMSO (Ctrl) or 1 μm palbociclib (Palbo). Quantification represents the percentage of cells positive for SA‐β‐gal staining (mean ± SEM of two independent experiments (*n* = 2) except for resistant cells and Meso56 (*n* = 1), as indicated below the graph). Scale bar, 100 μm. (D) Immunodetection of cleaved‐caspase 3 in cells grown for 3 days in the absence (Ctrl) or presence of 1 μm navitoclax (navito) or A1155463 (A11) and pre‐treated or not (Ctrl) for 9 days with 1 μm palbociclib (Palbo). *N* = 2 for H2052 and H28; *n* = 3 for H2452; *n* = 1 for Meso11, Meso13 and Meso56. *P < 0.05, **P < 0.01, ***P < 0.001, ****P < 0.0001 (Student's *t*‐test in A, ANOVA in B).

The effect of palbociclib on cell cycle progression was maintained for at least 2 weeks (Fig. 3A), contrary to the rapidly developing resistance to CDK4/6 inhibition described in breast cancer cells [80]. After 1 month of continuous treatment with palbociclib, some cell lines were still strongly inhibited whereas partial resistance arose in others (Fig. 3B). In sensitive MPM cells, prolonged treatment (9 days) with palbociclib induced a senescent phenotype characterized by an enlarged and flat cell morphology and by increased senescent‐associated β‐galactosidase activity (SA‐β‐gal) (Fig. 3C). Moreover, this long‐term pretreatment with palbociclib increased the apoptosis induced by senolytic drugs targeting Bcl‐2 and Bcl‐xL [86] (Fig. 3D).

**Fig. 4:**
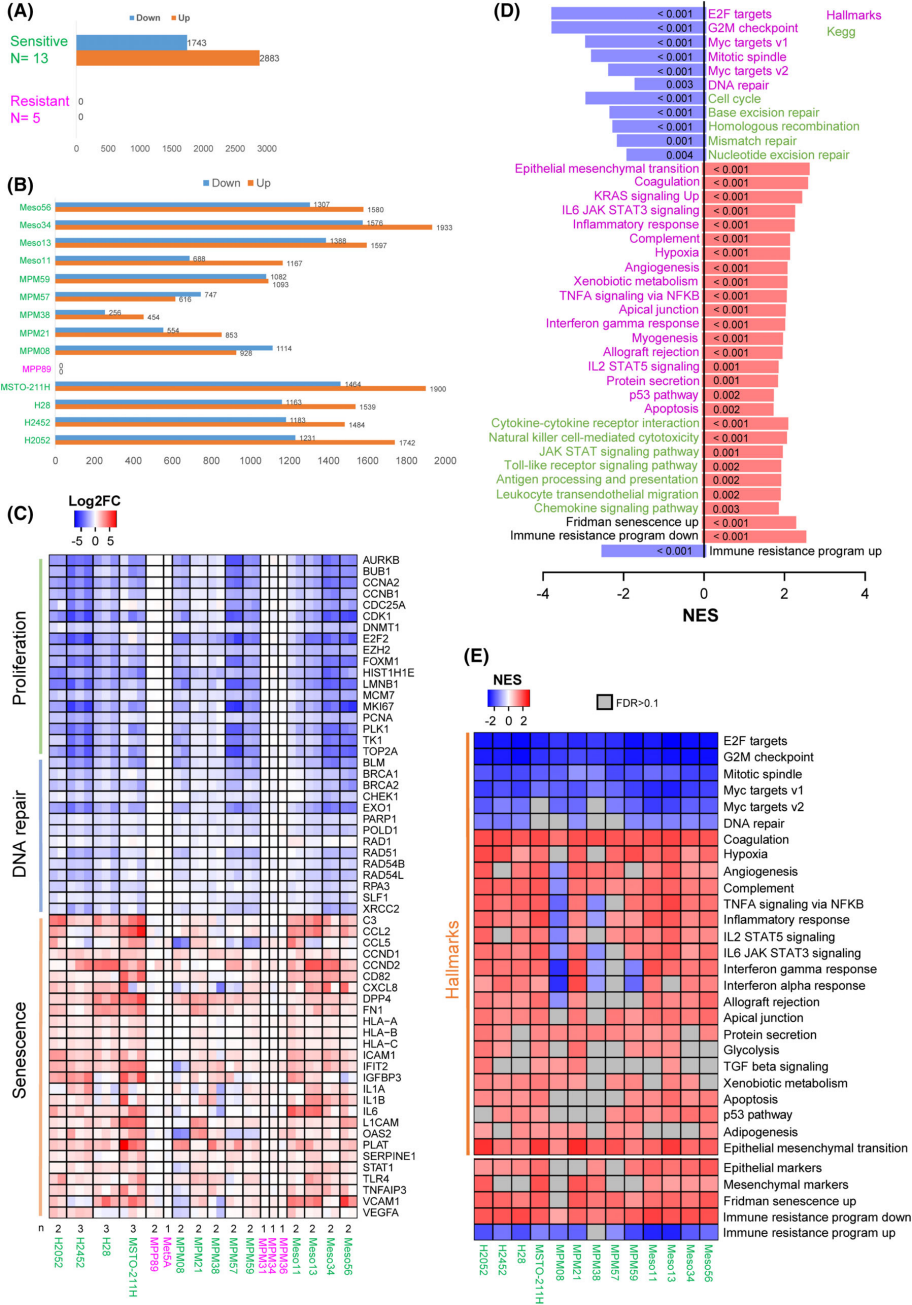
Impact of long‐term treatment with palbociclib analysed by RNA‐seq. 13 sensitive and five resistant cell lines were treated for 9–10 days with 1 μM palbociclib or DMSO. The number of independent experiments for each cell line is indicated in (C). Sensitive cell lines are in green, resistant ones are in magenta. (A) Number of genes down‐ or up‐regulated by palbociclib in a global analysis comparing sensitive and resistant cell lines. Differential expression analysis was performed using DESeq2 with min. fold change (FC) = 1.5 and false discovery rate (FDR) < 0.05. (B) Number of genes down‐ or up‐regulated by palbociclib for each cell line with minimum two independent experiments (min. FC = 1.5 and FDR < 0.05). (C) Heatmap representing genes involved in proliferation, DNA repair and senescence. Data are presented as log2FC (palbo/ctrl). *n* independent experiments are shown, as stated below the heatmap. (D) Gene Set Enrichment Analysis (GSEA) for palbociclib‐treated versus ctrl cells. Global analysis performed using all sensitive cell lines. Plot represents the normalized enrichment scores (NES) for the top regulated ‘Hallmarks’ pathways and related ‘Kegg’ pathways. ‘Fridman senescence up’ and gene sets up‐ or down‐regulated as part of an immune resistance programme are also illustrated. Down‐regulated pathways are in blue, up‐regulated pathways are in red. FDRs obtained for each pathway are indicated in the plot bars. (E) Heatmap illustrating the ‘Hallmarks’ pathways down‐ or up‐regulated by palbociclib in each sensitive cell line. Epithelial/ mesenchymal markers, ‘Fridman senescence up’ and gene sets up‐ or down‐regulated as part of an immune resistance programme are also shown. Plot represents the NES from GSEA. Pathways with FDR > 0.1 are in grey.

To further define the response of MPM cell lines to CDK4/6 inhibition, we compared the transcriptomic profiles of sensitive and resistant cell lines treated for 9–10 days with 1 μm palbociclib or DMSO (Fig. 4, Fig. S3). RNA‐seq data of selected targets were validated at the protein level by western blotting and the increased secretion of various components of the SASP was confirmed using cytokines arrays (Figs S4 and S5). Remarkably, no significant gene expression changes were observed in the insensitive cell lines (Fig. 4A,B), confirming the high specificity of this inhibitor used at 1 μm. This result also intriguingly indicated that both the absence of CDK4 phosphorylation in the presence of functional pRb (MPM_31 and MPM_34), and the loss of pRb in the presence of phosphorylated CDK4 (MPM_36), suffice to generate a complete insensitivity to palbociclib. In most sensitive cells, treatment with palbociclib induced a strong down‐regulation of genes (Fig. 4C; Fig. S3A) and proteins (Figs S4A,B and S5A,B) involved in cell cycle progression and DNA repair. Moreover, the long‐term treatment with the CDK4/6i increased the expression of many genes, the number of which even exceeded the number of down‐regulated ones (Fig. 4A,B; Tables S4 and S5). These included various genes and proteins classically involved in senescence (Fig. 4C; Figs S3A, S4 and S5), such as D‐type cyclins, cell adhesion molecules (ICAM1, VCAM1, L1CAM), and SASP components, such as SERPINs, tissue plasminogen activator (PLAT), fibronectin (*FN1*), IGFBPs, complement C3 and VEGFA. Several pro‐inflammatory cytokines (IL1A/B, IL6) and chemokines [CCL2, CCL5, CXCL8 (IL8)], as well as major histocompatibility complex class I, were also up‐regulated.

Gene Set Enrichment Analysis identified ‘E2F targets’ and ‘DNA repair’ among the top down‐regulated ‘Hallmarks’ pathways in response to CDK4/6 inhibition (Fig. 4D,E; Fig. S3B; Tables S6 and S7). Related ‘Kegg’ pathways including ‘Cell cycle’ and ‘Base excision repair’ were also strongly down‐regulated by palbociclib. On the other hand, signatures related to senescence and immune response such as ‘Fridman senescence up’, ‘TNFA signalling via NFKB’, ‘Cytokine–cytokine receptor interaction’, ‘Natural killer cell‐mediated cytotoxicity’, ‘JAK STAT signalling pathway’, ‘Antigen processing and presentation’, ‘Leukocyte transendothelial migration’ were enriched in palbociclib‐treated cells (Fig. 4D,E; Fig. S3B; Tables S6 and S7). ‘Interferon gamma response’ and ‘Interferon alpha response’ were also very significantly up‐regulated by palbociclib, but interestingly were not associated to a detectable expression of any type I, II or III interferon genes, consistent with the loss of their locus or low basal expression, as reported previously [57]. ‘Complement’, ‘Coagulation’, ‘Angiogenesis’ and ‘Apoptosis’ were also increased by palbociclib treatment. Of note, ‘Epithelial Mesenchymal Transition’ was the top up‐regulated ‘Hallmark’ pathway (Fig. 4D,E). However, this most likely reflected the profound remodelling of the extracellular matrix and cytoskeleton that is also associated with senescence. Indeed, palbociclib up‐regulated both ‘epithelial’ and ‘mesenchymal’ markers (defined as in ref. [59]), which is not consistent with EMT induction (Fig. 4E). Moreover, classical changes linked to EMT such as reduced E‐cadherin expression and up‐regulation of vimentin, N‐cadherin and several EMT transcriptional inducers were not observed, whereas some epithelial markers including *CLDN1* were up‐regulated in most cell lines (Fig. S3A). Analysis of enriched pathways in individual cell lines illustrated some heterogeneity in the pathways up‐regulated by palbociclib. It also highlighted an opposite regulation of the interferon signalling, in particular in MPM_08 cells that were characterized by high basal expression of *STAT1* and of interferon‐induced genes (*ISG*s) (Fig. 4E; Fig. S3C; Table S7). Most importantly, the inhibition of CDK4/6 was nevertheless able to reverse, in all the evaluated MPM cell lines, the ‘immune resistance programme’ that promotes T cell exclusion and resistance to immunotherapy in melanoma [66] (Fig. 4D,E).

### 3.4 Growth arrest induced by prolonged palbociclib treatment appears mostly irreversible

**Fig. 5:**
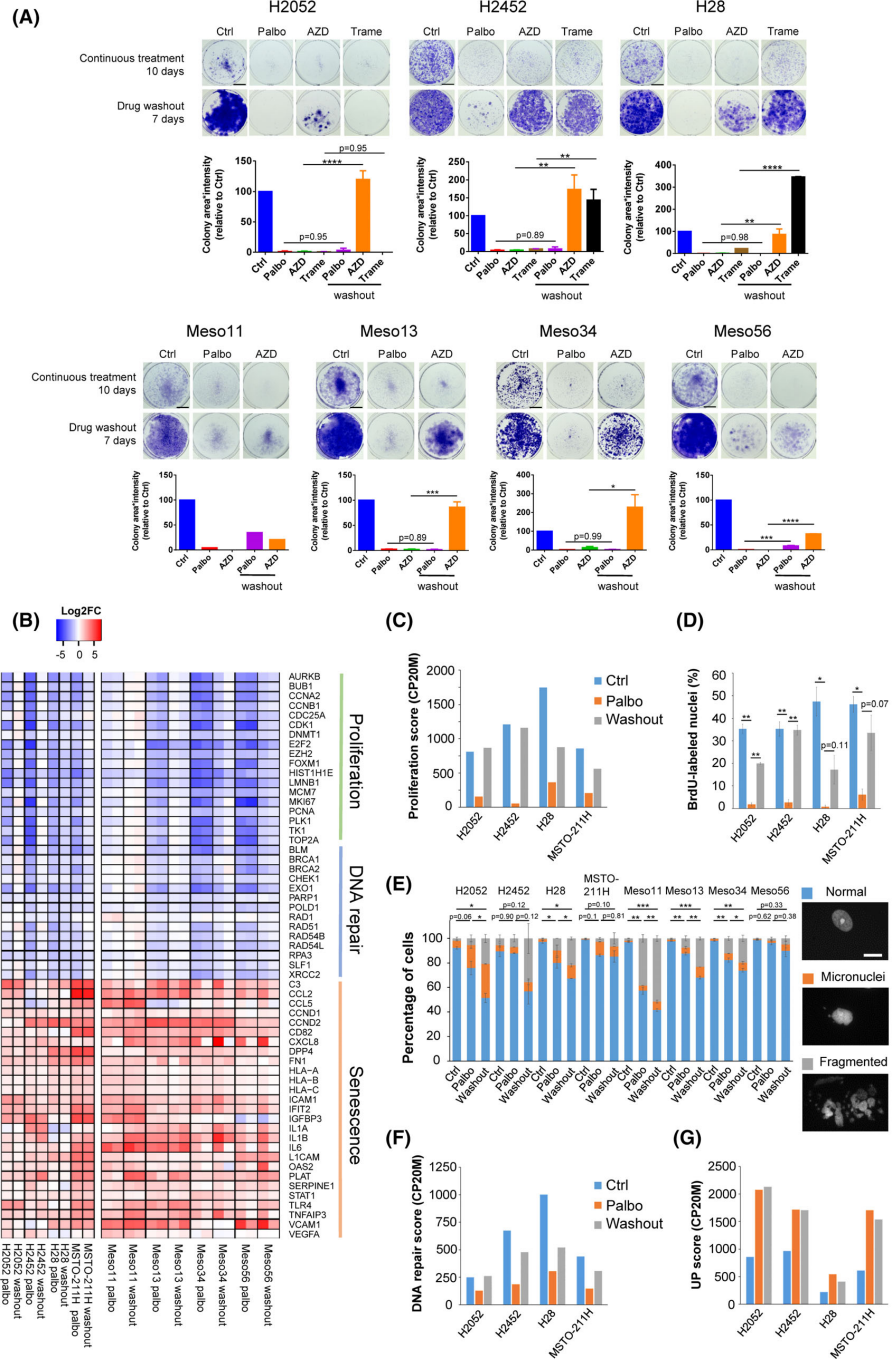
Palbociclib‐induced growth arrest is poorly reversible. (A) Clonogenic assays of cells treated continuously for 10 days with 1 μm palbociclib (Palbo), 50 nm of AZD8055 (AZD, mTOR inhibitor) or 20 nm trametinib (Trame, MEK inhibitor) followed or not by a drug washout for 7 days. Representative images are illustrated above the quantification of the staining using area * intensity expressed in percentage of control (Ctrl). Mean ± SEM of two independent experiments except for meso11 (*n* = 1). Scale bar, 1 cm. (B) Differential expression of genes involved in proliferation, DNA repair and senescence assessed by RNA‐seq in cells treated for 10 days with 1 μm palbociclib (Palbo) followed or not by a drug washout for 48 h. Data are presented as log2FC (Palbo/ctrl and washout/ctrl). Left part, *n* = 1. Right part, *n* = 2. (C) CCP proliferation score calculated from the RNA‐seq data illustrated in (B) and expressed in counts per 20 million reads (CP20M). (D) DNA synthesis in cells treated as in (B). Mean ± SEM of two independent experiments. (E) Percentage of normal nuclei, micronuclei or fragmented nuclei in cells treated as in (B). Mean ± SEM of two independent experiments. Statistical analysis was performed on the abnormal nuclei (micronuclei + fragmented nuclei). Representative images are shown on the right. Scale bars, 20 μm. (F) DNA repair score calculated from the RNA‐seq data and expressed in counts per 20 million reads (CP20M). This score represents the median expression of the genes involved in DNA repair illustrated in (B). (G) Expression score of up‐regulated genes calculated from the RNA‐seq data illustrated in Fig. S6A and expressed in counts per 20 million reads (CP20M). (A, D, E) *P < 0.05, **P < 0.01, ***P < 0.001, ****P < 0.0001 (ANOVA).

In clinics, palbociclib and ribociclib are administered discontinuously with 1 week off treatment every 3 weeks. Therefore, we evaluated the effect of palbociclib washout and the reversibility of the cell cycle arrest induced by this drug. When assessed by clonogenic assay, the effect of a 10‐day treatment with palbociclib was mostly irreversible. This contrasted with the reversible growth arrest observed with AZD8055 and trametinib (mTOR and MEK inhibitors respectively) for most of the investigated cell lines (Fig. 5A). However, 48 h after palbociclib washout, proliferation genes were re‐induced (Fig. 5B; Fig. S6A), which was associated with the increase of a proliferation score calculated from RNA‐seq data based on the ‘Cell Cycle Proliferation’ (CCP) signature [67] (Fig. 5C, Fig. S6B). The phosphorylation of pRb also increased after drug withdrawal (Fig. S4B) and cells were able to re‐enter S‐phase (Fig. 5D, Fig. S6C). Nevertheless, this cell cycle re‐entry might be abortive, as cells were unable to actively proliferate in the colony‐forming assay. As thoroughly investigated in a recent study [87], this could be due to the accumulation of abnormal nuclear figures (micronuclei, fragmented nuclei) observed after palbociclib treatment and even more after withdrawal of the drug (Fig. 5E). This was associated with an increase of DNA damage illustrated by γH2AX staining during palbociclib treatment, which was maintained after drug washout (Fig. S6F). Interestingly, whereas the repression of E2F‐dependent cell cycle and DNA repair genes was essentially reversible, most gene up‐regulations including those associated with SASP and productions of various cytokines, were maintained or even more augmented after palbociclib washout (Fig. 5B,F,G; Fig. S4B,D, S5 and S6A,E).

### 3.5 CDK4 phosphorylation is detected in a majority of MPM tumours

**Fig. 6:**
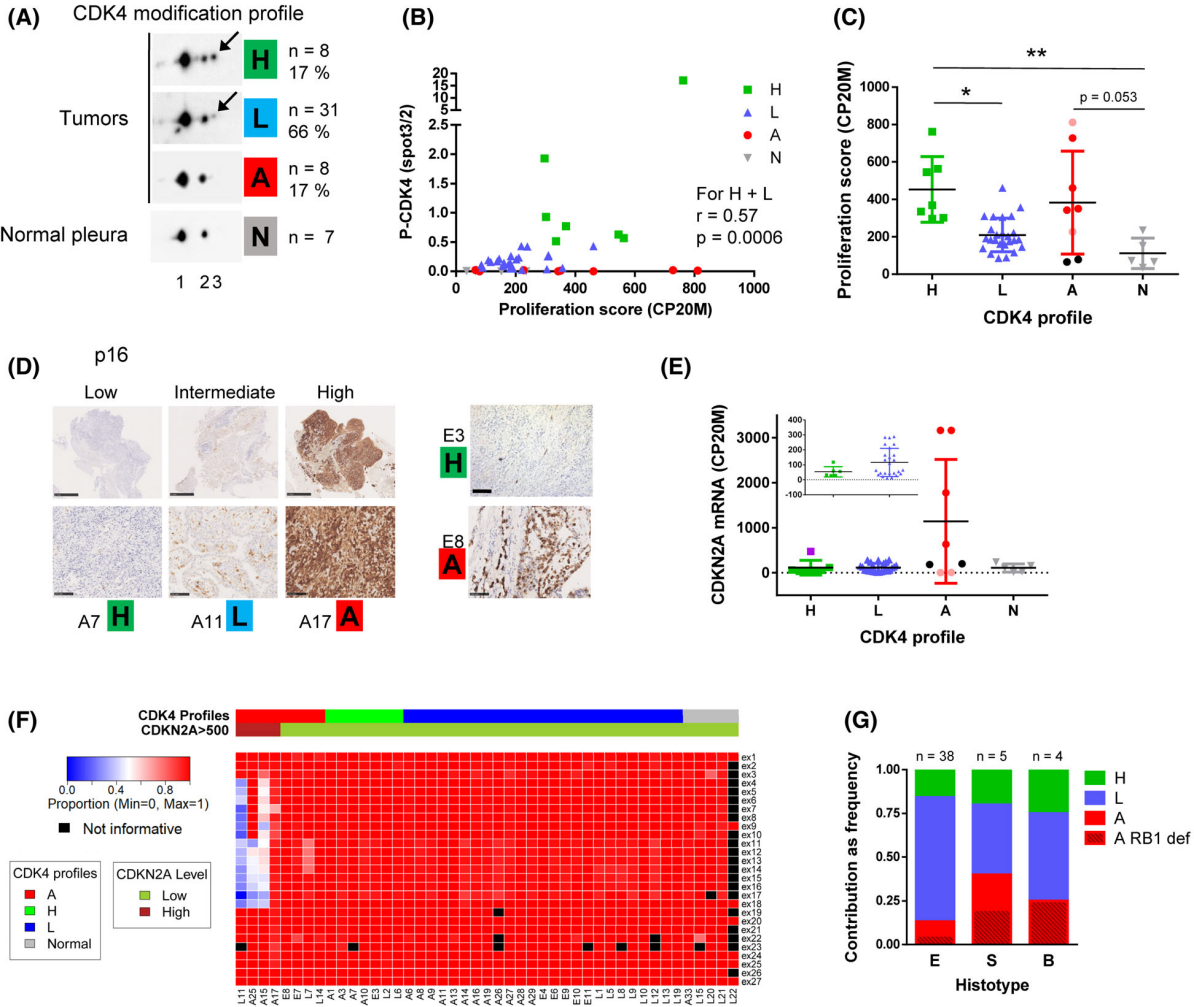
CDK4 phosphorylation is detected in a majority of MPM tumours. (A) Representative immunodetections of CDK4 separated by 2D‐gel electrophoresis from 47 tumours and seven normal pleurae. A CDK4 modification profile was attributed to each tumour based on the ratio (r) spot3/spot2 quantified from the immunoblots. High (H): *r* > 0.5, Low (L): 0.025 ≤ *r* ≤ 0.5, Absent (A): *r* < 0.025. The number and percentage of samples in each class is indicated on the right. Arrows indicate the position of the T172‐phosphorylated form of CDK4 (spot 3). (B) Phosphorylation of CDK4 (ratio spot3/spot2) as a function of the CCP proliferation score calculated from RNA‐seq data (*n* = 40). Correlation between CDK4 phosphorylation and proliferation for H and L profiles was evaluated with a Spearman's rank test. (C) Proliferation score in the different classes of tumours (H, L and A) and in normal pleura (N). For profiles A: red dots = absence of CDK4 phosphorylation with high *CDKN2A* expression and high proliferation score, pink dots = absence of CDK4 phosphorylation with low *CDKN2A* expression and high proliferation score, black dots = absence of CDK4 phosphorylation with intermediate *CDKN2A* expression and low proliferation score. Mean ± SD, *P < 0.05, **P < 0.01, Kruskal–Wallis followed by Dunn's multiple comparison tests. (D) Representative IHC staining of p16. H, L, A, CDK4 modification profile. Scale bars, 100 μm except for the three left upper panels (1 mm). p16 staining was performed once for each tumour sample (*n* = 1). (E) *CDKN2A* mRNA levels (RNA‐seq data in CP20M) in the different classes of tumours and in normal tissues. For class A tumours, dots colour like in (C). In class H tumours, the purple square represents the E3 sample that presents relatively high levels of *CDKN2A* together with low p16 staining associated with a *CDKN2A* mutation. Error bars: mean ± SD. (F) Heatmap representing the proportion of correctly spliced exons in *RB1* calculated from RNA‐seq data for 40 tumours and five normal pleurae. Tumours are grouped by CDK4 modification profile and separated based on *CDKN2A* expression. Values were considered not informative when < 10 reads were covering the region. (G) Relative proportions of the three CDK4 modification profiles in the different histotypes. E: epithelioid, S: sarcomatoid, B: biphasic. Class A samples with *RB1* defect are distinguished by a striped pattern. The number of samples in each histotype is indicated at the top of each bar. In A and G: *n* = 8 for profile H, *n* = 31 for profile L and *n* = 8 for profile A. In B, C, E and F: *n* = 7 for profile H, *n* = 25 for profile L, *n* = 8 for profile A and *n* = 5 for normal pleura.

To evaluate the proportion of MPM patients that would be potentially responsive or intrinsically resistant to CDK4/6 inhibition, we determined the CDK4 modification profile from frozen MPM tumours and normal pleura samples (Table S8A) as investigated previously in breast cancer [51]. Whereas it was absent in normal pleura, CDK4 phosphorylation was detected with variable abundance in 39 of 47 tumours, suggesting that about 80% of patients could respond to CDK4/6 inhibition (Fig. 6A; Fig. S7). Tumours were classified as done previously [51] in three groups based on their CDK4 phosphorylation status: A (absence of phosphorylation), L (low phosphorylation) and H (high phosphorylation). When CDK4 phosphorylation was detected, its relative abundance significantly correlated with the ‘Cell Cycle Proliferation’ score calculated from RNA‐seq data (Fig. 6B), which was significantly different in class H versus class L tumours (Fig. 6C). Two samples with no CDK4 phosphorylation (E8, L7) were not proliferative (Fig. 6C, Table S8A) and stained positive for p16 (Fig. 6D; Table S8A), indicating that these tumour samples could be in a senescent state [88]. However, these two patients did not receive any chemotherapy before the biopsy. We also note that they are long‐term survivors with one patient having a 13‐year survival and the other being alive more than 6 years after diagnosis (Table S8A). Unexpectedly, CDK4 phosphorylation was undetectable in six proliferative tumours. For four of them, this was associated with an extremely high expression of p16 at both protein (Fig. 6D; Table S8A) and RNA (Fig. 6E; Table S8B) levels. Interestingly, the tumour with the highest degree of CDK4 phosphorylation (E3, a patient who died 2 months after diagnosis) presented also an elevated *CDKN2A* mRNA expression (Fig. 6E; Table S8B), but its p16 staining was weak (Fig. 6D; Table S8A). This was associated with a partial *CDKN2A* deletion affecting the exon 3 (Table S8B). High p16 expression is often associated with an inactivation of pRb/*RB1*. However, the p16‐high class A tumours were not distinguished by low *RB1* expression (Fig. S8A) and no mutation (substitution or Indel) was detected using the RNA‐seq data (Table S8B). pRb inactivation by SV40 large T [76] was also unlikely (Table S3). RNA‐sequencing rather revealed splicing alterations (Fig. 6F). Targeted DNA‐sequencing confirmed the absence of *RB1* mutations and demonstrated the loss of one *RB1* allele in at least three of these four samples (Fig. S8B,C). Moreover, different partial deletions of *RB1* were found in the remaining allele (Fig. S8D,E). Our data thus suggest a double hit inactivation of the *RB1* gene in the p16‐high class A tumours. Surprisingly, phospho‐CDK4 was also undetectable in two proliferative tumours expressing very low levels of *CDKN2A* (E7 and L14, Fig. S7 and Table S8B). Even though we cannot totally exclude a loss of CDK4 phosphorylation during sample processing, these two samples could represent particular class A tumours. E7 tumour has the highest expression of *CDK6* associated with high levels of *CDKN2C* (Table S8B). This suggests it could depend on CDK6 instead of CDK4 and therefore be resistant to palbociclib, since CDK6 overexpression is a well‐known mechanism of resistance to CDK4/6 inhibition [83, 85] (although we did not observe resistance to palbociclib in MPM cell lines that express the highest CDK6 levels). CDK6 up‐regulation has been shown to induce *CDKN2C* encoding p18 that binds to CDK4/6, potentially preventing phosphorylation of these kinases and also competing with CDK4/6 inhibitory drugs [83]. L14 tumour also expressed high levels of *CDK6* and *CDKN2C*. That, together with a low *CCND1* expression, could explain the absence of phosphorylated CDK4 in this sample. Finally, profile A tumours, including those with *RB1* deletion, were observed in the different histotypes (Fig. 6G).

### 3.6 High expressions of *CDKN2A* and/or *CCNE1* are observed in the presence or absence of *RB1* defect and are associated with a shorter overall survival in MPM patients

**Fig. 7:**
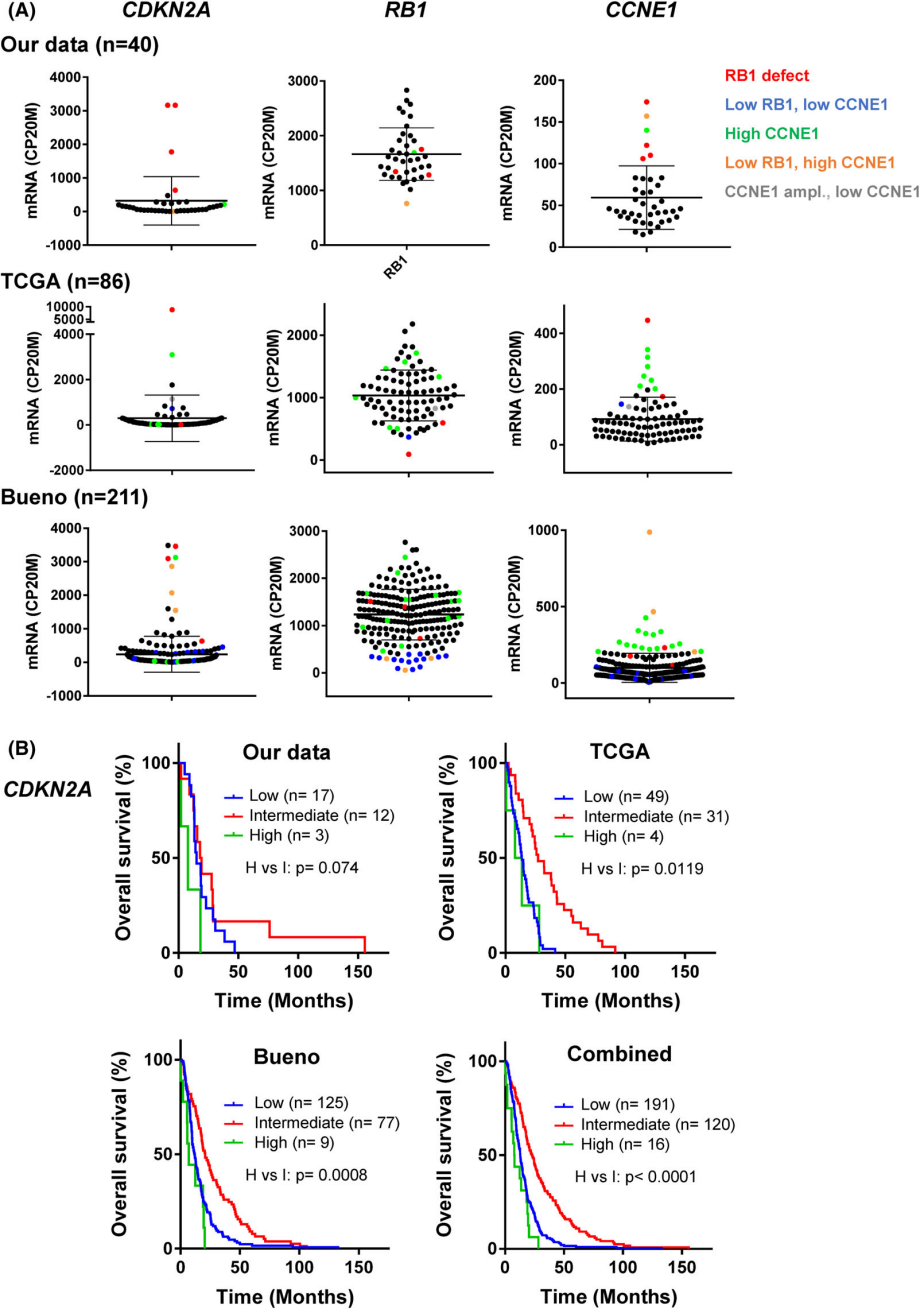
High expressions of *CDKN2A* and/or *CCNE1* are associated with a shorter overall survival in MPM patients. (A) *CDKN2A*, *RB1* and *CCNE1* mRNA expression in our data (*n* = 40) and in the TCGA (*n* = 86) and Bueno et al. (*n* = 211) cohorts. RNA‐seq data were normalized relative to the library size in counts per 20 million reads (CP20M). Error bars: mean ± SD. Colour code, red: *RB1* defect (homozygous deletion or mutation), blue: low *RB1* expression with low *CCNE1* level, orange: low *RB1* expression with high *CCNE1* level, green: high *CCNE1* expression without *RB1* defect or low expression, grey: *CCNE1* amplification without high expression. (B) Kaplan–Meier curves comparing overall survival of patients with high, intermediate or low *CDKN2A* expression in our data (*n* = 32), the TCGA (*n* = 84) and Bueno et al. (*n* = 211) cohorts; or in the merged combination of the three cohorts (*n* = 327). *P*‐values were calculated using a log‐rank test.

Based on our observations in MPM tumours and cell lines, we next evaluated the occurrence of high *CDKN2A* and *CCNE1* expressions and their relationship with *RB1* defects using publicly available RNA‐seq data from cohorts of the Bueno et al. study (*n* = 211) [12] and The Cancer Genome Atlas (TCGA‐Meso, *n* = 86). Merging these two cohorts and our own RNA‐seq data, high *CDKN2A* transcript levels were observed in a total of 17 of 141 (12 %) MPMs expressing *CDKN2A* (which represented 5% of the overall 331 patients; Fig. 7A; Table S9). Such high *CDKN2A* expressers were found in the three main MPM histotypes (Fig. S9A). Most tumours with previously identified *RB1* defects were associated with very high *CDKN2A* transcript levels as expected, which were also observed in some tumours with very low *RB1* transcript levels (Fig. 7A; Table S9). Nevertheless, seven tumours were observed with defects or very low expression of both *RB1* and *CDKN2A*, showing that the coexistence of both defects in MPM_36 cells might not result from a culture artefact. Both in Bueno and TCGA cohorts, we observed that several tumours with high *CDKN2A* transcripts did not display any defect or low expression of *RB1*. Some of these tumours were associated with amplification or overexpression of *CCNE1*, as we characterized it in MPM_31 and MPM_34 cell lines; and/or with high transcript levels of E2F1, or E2F3 in the peculiar M631PT patient in Bueno's cohort (Fig. 7A; Table S9).

Deletion or low expression of *CDKN2A* are known to be associated with a shorter overall survival in MPM patients [8, 15, 52]. When stratification is based on median expression, patients with higher expression of *CDKN2A* have thus a better outcome [52]. We wondered whether the tumours that express especially high p16/*CDKN2A* levels, as we observed them in CDK4 profile A tumours, might be associated with a different outcome. Indeed, similar to MPM patients with low *CDKN2A*, patients with very high *CDKN2A* expression had a much shorter survival than those with an intermediate expression in the three cohorts (7.88 vs. 23.10 months in the combined cohort, HR = 0.31, CI = 0.05–0.28). We also observed a shorter overall survival in patients with *CCNE1* overexpression (Fig. S9B).

## 4 Discussion

In this work, we have evaluated the effect of CDK4/6i in a panel of 28 MPM cell lines including 19 patient‐derived cell lines from two independent collections. Extending the observations by Bonelli et al. [53] and Aliagas et al. [52], we found that the majority of these models were particularly sensitive to CDK4/6 inhibition, irrespective of their histotype. Indeed, in most cell lines, continuous palbociclib treatment produced an almost complete cell cycle inhibition, which was sustained for at least 2 weeks. This prolonged treatment irreversibly inhibited the proliferation in colony‐forming assay, despite re‐induction of pRb phosphorylation and cell cycle genes upon drug washout. A SASP including various potentially immunogenic components was also irreversibly induced. This remarkable sensitivity of MPM cell lines to CDK4/6 inhibition contrasts with a more partial response and/or rapidly developing resistance (adaptation) observed in other cancers such as oestrogen receptor‐positive breast cancer [80], pancreatic ductal adenocarcinoma (PDAC) [89, 90], colorectal cancer [91] and anaplastic thyroid cancer [92]. In those tumours, combined inhibition of CDK4/6 and signalling pathways upstream of CDK4 activation (MAPK and/or PI3K‐mTOR [50]) is required to fully and durably suppress their proliferation [80, 89, 90, 93, 94]. Such an exquisite sensitivity to CDK4/6 inhibition might be explained by the absence of oncogenic driver mutations affecting those signalling cascades in mesotheliomas, which are mostly characterized by the inactivation of tumour suppressor genes [8, 12, 13, 14].

On the other hand, four MPM cell lines and immortalized MeT‐5A cells were completely insensitive to CDK4/6i, with no inhibition of cell cycle progression and no induction of SASP transcriptional responses. For most of these cell lines, the insensitivity to palbociclib was associated with their lack of T172‐phosphorylation of CDK4 and high p16 accumulation that diverted CDK4 from binding D‐type cyclins. However, *RB1* defects could neither be detected by RNA‐seq nor by analyses of pRb accumulation and phosphorylation in the patient‐derived cell lines MPM_31 and MPM_34. Therefore, superior to pRb, the status of CDK4 phosphorylation was able to predict the response to palbociclib in 27/28 mesothelial cell lines, as we also observed it in breast cancer [51]. The involvement of p16 is further supported by the observation that the only resistant cell line displaying some CDK4 phosphorylation (MPM_36) combines a deletion of *RB1* with the loss of *CDKN2A*. Among other causes [95], p16 overexpression in cancer cells has been related to *RB1* loss of function [74, 75], which prevents the *CDKN2A* locus silencing by Polycomb Repressor Complexes [96, 97] and trimethylation of lysine 27 on histone H3 by EZH2 [98, 99]. This silencing might be directed by interaction of EZH2 with pRb and E2F1 [100]. Interestingly, our data suggest that high levels of p16/*CDKN2A* could also be associated with constitutive overactivation of CDK2 in MPM_31 and MPM_34 cells, resulting from amplification of *CCNE1* and possibly associated with other mechanisms including a p27/*CDKN1B* mutation or high cdc25 phosphatase activity (Fig. 2E–G). This overactivation of CDK2 can result in constitutive inactivation of pRb by phosphorylation. Alternatively, we do not exclude that these high p16 levels could be the consequence of replicative stress [101], which might be provoked by deregulated CDK2 activity. Indeed, both *CCNE1* overexpression and *RB1* alterations have been shown to induce the DNA Damage Response (DDR) [102]. Our results thus suggest that in cells with wild‐type *CDKN2A*, the insensitivity to CDK4 inhibition could be directly due to the absence of the main target of CDK4/6i, that is, active phosphorylated CDK4, rather than to the two generally considered mechanisms—absence/mutation of pRb or amplified cyclin E1‐mediated activation of CDK2 [36, 80, 81, 82, 103, 104]. It remains difficult to understand why the deficiency of pRb is sufficient to generate the observed insensitivity to CDK4/6i in the presence of the other proteins of the RB family, p107 (*RBL1*) and p130 (*RB2*), which are also inactivated by CDK4 phosphorylation and capable of blocking the cell cycle [38, 39, 40, 41, 105, 106]. The absence of CDK4 phosphorylation is a more likely explanation for a complete insensitivity to CDK4 inhibitors. Indeed, as the T172‐phosphorylation is required for the opening of the catalytic site of CDK4 [107, 108], it is not only critical for the activity of CDK4 complexes, but also potentially required for the engagement of CDK4 by ATP‐competitive inhibitors like palbociclib. This likely explains why in RB‐deficient models expressing high p16, palbociclib is observed not to bind to CDK4 [109, 110]. The lack of activating T177‐phosphorylation in CDK6, which is more frequent [46], may also explain the weak binding of CDK4/6i to CDK6 in the resistant cells that overexpress it [83, 84]. Indeed, the sensitivity to CDK4/6i was restored by the S178P mutation of CDK6 [84] that forces its T177‐phosphorylation [46].

We detected CDK4 phosphorylation in 80% of MPM tumours suggesting that the majority of the patients could at least initially respond to CDK4/6i. Treatment of relapsed mesothelioma with abemaciclib has resulted in 15% partial responses and 54% stable diseases in a recent phase II clinical trial [NCT03654833] using negative p16 IHC staining as an inclusion criterion [54]. However, as initially observed in a subset of breast cancers [51] and here in most insensitive MPM cell lines, 15% of MPM tumours were highly proliferative despite the lack of CDK4 phosphorylation and thus are expected to be intrinsically resistant to CDK4/6i. For two‐thirds of them, this was associated with copy number loss and/or partial deletions of *RB1* leading to a high p16/*CDKN2A* expression, which has also been observed by others in 17% of 88 MPMs [111]. Whether such elevated p16 accumulations might be associated to previously unrecognized *RB1* alterations including some monoallelic deletions, which were reported to be relatively frequent (up to 25%) [17, 112], or other causes such as inactivation of pRb by CDK2‐dependent phosphorylation, should be evaluated in other MPM cohorts. Indeed, our further exploration of public Bueno and TCGA datasets demonstrated that especially high *CDKN2A* transcript levels occurred in a subset (12%) of *CDKN2A* expressing tumours that were associated with poorer survival. This subset included not only the very few cases with biallelic *RB1* defects but also other MPMs harbouring either low *RB1* expression or overexpression of *CCNE1* or *E2F* in the presence of normal expression of wt *RB1*, as evidenced and characterized in MPM_31 and MPM_34 cell lines.

*RB1* status has been shown to govern differential sensitivity to genotoxic and molecularly targeted therapeutic agents, *RB1* defects generally sensitizing to the formers [113, 114]. Interestingly, in tumours predicted to be insensitive to CDK4/6i, transient administration of these drugs could be used to transiently arrest proliferation of normal cells and prevent chemotherapy‐induced myelosuppression, hence allowing to increase the dose of genotoxic drugs such as cisplatin and thus the therapeutic window between normal and transformed cells [115]. This idea has been validated clinically in small cell lung cancers that are generally insensitive to CDK4/6i due to their *RB1* locus alteration [116], leading to recent approval by the FDA of trilaciclib for this indication. Therefore, defining or predicting the CDK4 phosphorylation status might really be one key to tailor the use of CDK4/6i in MPM treatment and their combination with targeted or genotoxic therapies.

If frozen tissue samples are available, the presence or absence of phosphorylated CDK4 could be the most direct biomarker to predict sensitivity to CDK4/6i. However, in FFPE samples, the IHC detection of phosphorylated CDK4 would be complicated by its extremely low abundance [117] and by the loss of phosphorylation events before and during formalin fixation. Gene expression signatures including from RNA‐seq data might instead be used to predict the CDK4 status [51]. Since most MPM tumours lacking phospho‐CDK4 had high p16 expression, appropriately scored p16 immunohistochemistry (especially regarding intensity and homogeneity of expression [75, 111]), possibly in combination with proliferation markers, could also be used to exclude those intrinsically resistant tumours.

As characterized in some other cancers [21, 27, 32, 118], our present data suggest that MPM might be especially responsive to various (nonexclusive) combinatorial approaches to extend the cytostatic activity of CDK4/6i into a real clearance and eradication of tumour cells:

1. *Combination with senolytics*. Senescent cells harbour vulnerabilities allowing their specific killing by various ‘senolytics’ [24, 119], including BCL‐2 and BCL‐xL inhibitors (venetoclax, navitoclax, A1331852, …) that were observed to augment the tumour response to CDK4/6i [120, 121]. Prolonged palbociclib treatment indeed increased transcriptomic pathways related to apoptosis in nine of 13 sensitive MPM cell lines and apoptotic cell death induced by the BCL inhibitors Navitoclax and A1155463 (Fig. 3C). Further studies should evaluate new generations of such drugs, for example, recently described BCL‐XL PROTAC degraders [122] or galacto‐conjugated navitoclax prodrug to specifically target the SAβGal‐positive senescent cells [123].
2. *Combinations of CDK4/6i with chemotherapy*. CDK4/6 inhibition, by blocking cells into G1 phase, has been generally thought to antagonize the effect of cell cycle‐dependent chemotherapy. Nevertheless, a number of more recent preclinical studies also suggested that the simultaneous combination of chemotherapy and CDK4/6 inhibition can have cooperative antitumour effects, in part because CDK4/6 inhibition also reduces the E2F‐dependent expression of multiple genes required for DNA damage repair and thus would limit the ability of tumour cells to recover from chemotherapy‐mediated damage [27, 28]. Several observations in the present study together with recent reports suggest that both sequential and simultaneous combinations should be evaluated in further MPM studies: (a) in clinics, palbociclib and ribociclib are used discontinuously with 1 week off treatment every 3 weeks. We observed that palbociclib paradoxically stabilizes p21/p27‐free cyclin D‐CDK4/6 complexes that become hyperactive upon palbociclib withdrawal, potentially inducing a burst of tumour cell cycle progression that might open a window of increased responsiveness to genotoxic therapies [64]. As thoroughly investigated in a recent study [87], we observed here that following a prolonged G1‐arrest, the cell cycle entry induced by palbociclib washout is clearly perturbed, as it did not lead to clonogenic proliferation but to various potentially stressful nuclear defects that are associated with a sustained increase of DNA damage illustrated by γH2AX staining; (b) we observed that palbociclib down‐regulates various DNA repair genes and pathways in MPM cells, which would favour a cooperation between CDK4/6i and DNA repair inhibitors such as PARP inhibitors (olaparib) [87, 124, 125, 126]; (c) therapy‐induced SASP could increase delivery of chemotherapy by inducing a vascular remodelling [127], which might also occur in CDK4/6i‐treated MPM, as suggested in our data by the up‐regulation of angiogenesis pathway and VEGF in nine of 13 sensitive MPM cell lines.
3. *Combination of CDK4/6i with immunotherapy*. Inhibition of CDK4/6 has been shown to favour the elimination of tumour cells by the adaptive immune system through various mechanisms including enhanced antigen presentation by cancer cells, increased infiltration and activation of T cells; and inhibition of regulatory T cells proliferation [29, 30, 128, 129, 130]. On the other hand, therapy‐induced senescence was also shown to elicit a distinct mechanism of innate immune attack by NK cells, which is mediated by an NF‐kB‐dependent SASP programme that culminates in the secretion of pro‐inflammatory cytokines and surface expression of NK cell‐activating molecules such as ICAM‐1 [90]. In our RNA‐seq data, pathways related to immune response and antigen presentation were among the top up‐regulated ones in response to palbociclib. CDK4/6 inhibition was associated with induction of a SASP of variable composition in the different cell lines. This included ICAM‐1 and pro‐inflammatory cytokines/chemokines such as IL6/CCL2/CCL5, known to promote the recruitment of various immune cells [26, 90, 131]. Moreover, we observed that CDK4/6 inhibition represses a cancer cell programme shown to mediate resistance to anti‐PD1 therapy in melanoma, consistent with studies demonstrating a cooperation between CDK4/6i and immune checkpoint blockade [66, 132]. Interestingly, after palbociclib washout most gene up‐regulations associated with SASP including productions of various cytokines were maintained or even more augmented (at variance with cell cycle genes), as also recently observed by others [131]. Therefore, this raises the plausible expectation that the drug‐holiday periods of patient treatments with palbociclib and ribociclib, in complement of inducing important stressful nuclear perturbations associated to unbalanced re‐induction of cell cycle genes, would sustain or enhance immune responses against tumour cells generated by the various SASP components. Overall, CDK4/6i, in addition to their direct cytostatic tumour action, would greatly facilitate response of MPMs to immune checkpoint inhibitors including the newly FDA‐approved combination of nivolumab and ipilimumab [2, 11, 132].

## 5 Conclusions

To conclude, our study supports further clinical evaluation of CDK4/6i for the treatment of pleural mesotheliomas including in various combinations with the standard therapies. Most MPM patients could respond to CDK4/6i, which may not only arrest tumour growth but also help to convert the mesotheliomas that are rarely immunologically ‘cold’ but more frequently in intermediate inflammatory states [133], into ‘hot’ tumours responsive to immunotherapy. Nevertheless, a minority of intrinsically CDK4/6i insensitive tumours lacking CDK4 phosphorylation and associated with poor survival might have to be identified.

## Conflict of interest

The authors declare no conflict of interest.

## Author contributions

PPR and ER conceived the project; SP, CM and JBA designed and performed experiments; PPR, ER, SP, XB, CM, CB and DJ analysed and discussed the data; ER, MT, YB and FL performed bioinformatics analyses; CB and DJ provided patient‐derived cell lines; MR, PP, NLS, STE, FGS, JPVM and TB provided MPM samples and related data; SP and PPR wrote the original draft. All the authors contributed to manuscript editing and approved the final version.

### Peer review

The peer review history for this article is available at https://publons.com/publon/10.1002/1878‐0261.13351.

## Supporting information


**Fig. S1.** Sensitivity of MPM cell lines to palbociclib is associated with CDK4 phosphorylation.
**Fig. S2.** Absence of CDK4 phosphorylation in resistant cells is due to high p16 levels associated or not with pRb defect.
**Fig. S3.** Impact of long‐term treatment with palbociclib analysed by RNA‐seq.
**Fig. S4.** Validation of RNA‐seq data at the protein level.
**Fig. S5.** Concordance between RNA‐seq data and proteic analyses.
**Fig. S6.** Effect of palbociclib washout.
**Fig. S7.** CDK4 phosphorylation is present in most MPM tumours.
**Fig. S8.** Double hits in *RB1* in high p16 expressing tumours: loss‐of‐heterozygosity and aberrant splicing.
**Fig. S9.** High expression of *CCNE1* is associated with a shorter overall survival in MPM patients.

**Table S1.** Drugs and antibodies.
**Table S2**. Cell lines characteristics.
**Table S3.** Quantification of viral genomes from RNA‐seq data.
**Table S4.** Genes down‐ or up‐regulated after long‐term treatment with palbociclib (global analysis).
**Table S6.** Pathways down‐ (A) or up‐regulated (B) after long‐term treatment with palbociclib in a global GSEA analysis.
**Table S8**. Tumours characteristics. Clinical data (A) and RNA‐seq data (B).
**Table S9**. Characteristics of the tumours from the TCGA‐Meso cohort (A) and from the Bueno et al. cohort (B).

**Table S5.** Genes down‐ or up‐regulated after long‐term treatment with palbociclib (analysis by cell line).

**Table S7.** Pathways deregulated after long‐term treatment with palbociclib in each cell line.

## Data Availability

The RNA‐sequencing data from commercial cell lines and the SNP array data from patient‐derived primary cell lines have been deposited in the Gene Expression Omnibus with accession number: GSE195568 and GSE197288 respectively. The RNA‐sequencing data from patient‐derived cell lines and the data from MPM patients has been deposited at the European Genome‐phenome Archive (EGA), which is hosted by the EBI and the CRG, under accession number EGAS00001006117. The TCGA data are available from the GDC portal (TCGA‐MESO cohort) and the data from the Bueno et al. cohort are available from EGA under accession number EGAS00001001563.
