# Supplementary material for: Preclinical evaluation of CDK4 phosphorylation predicts high sensitivity of pleural mesotheliomas to CDK4/6 inhibition
Source: Mol Oncol. 2022 Dec 19;18(4):866–94. doi: 10.1002/1878-0261.13351 (PMC10994244; doi:10.1002/1878-0261.13351)
Supplement: Supplementary file 1 — Fig. S1. Sensitivity of MPM cell lines to palbociclib is associated with CDK4 phosphorylation. Fig. S2. Absence of CDK4 phosphorylation in resistant cells is due to high p16 levels associated or not with pRb defect. Fig. S3. Impact of long‐term treatment with palbociclib analysed by RNA‐seq. Fig. S4. Validation of RNA‐seq data at the protein level. Fig. S5. Concordance between RNA‐seq data and proteic analyses. Fig. S6. Effect of palbociclib washout. Fig. S7. CDK4 phosphorylation is present in most MPM tumours. Fig. S8. Double hits in RB1 in high p16 expressing tumours: loss‐of‐heterozygosity and aberrant splicing. Fig. S9. High expression of CCNE1 is associated with a shorter overall survival in MPM patients. [file MOL2-18-866-s003.pdf]

Supplementary information

Preclinical evaluation of CDK4 phosphorylation predicts high sensitivity of pleural mesotheliomas to CDK4/6 inhibition

Sabine Paternot[1§], Eric Raspé[1#], Clément Meiller[2#], Maxime Tarabichi[1], Jean-Baptiste Assié[2,3,4], Frederick Libert[1,5], Myriam Remmelink[6], Xavier Bisteau[1], Patrick Pauwels[7,8], Yuna Blum[9,10], Nolwenn Le Stang[11], Séverine Tabone-Eglinger[11], Françoise Galateau-Sallé[11,12], Christophe Blanquart[13], Jan P. Van Meerbeeck[14], Thierry Berghmans[15], Didier Jean[2], Pierre P. Roger[1§]*.

[1] Institut de Recherche Interdisciplinaire en Biologie Humaine et Moléculaire (IRIBHM), Université Libre de Bruxelles, Brussels, Belgium. ULB-Cancer Research Center (U-CRC), Université Libre de Bruxelles, Brussels, Belgium.

[2] Centre de Recherche des Cordeliers, Inserm, Sorbonne Université, Université de Paris, Functional Genomics of Solid Tumors, Paris, France.

[3] University Paris-Est Créteil (UPEC), CEpiA (Clinical Epidemiology and Ageing), EA 7376-IMRB, UPEC, Créteil, France.

[4] GRC OncoThoParisEst, Service de Pneumologie, CHI Créteil, UPEC, Créteil, France.

[5] BRIGHTCore, ULB, Brussels, Belgium.

[6] Department of Pathology, Erasme Hospital, Université Libre de Bruxelles, Brussels, Belgium.

[7] Center for Oncological Research (CORE), Integrated Personalized and Precision Oncology Network (IPPON), Wilrijk, Belgium.

[8] Department of Pathology, Antwerp University Hospital, Edegem, Belgium.

[9] Programme Cartes d'Identité des Tumeurs (CIT), Ligue Nationale Contre Le Cancer, Paris, France.

[10] Present address: IGDR UMR 6290, CNRS, Université de Rennes 1, Rennes, France.

[11] MESOBANK, Department of Biopathology, Centre Léon Bérard, Lyon, France.

[12] Cancer Research Center INSERM U1052-CNRS 5286R, Lyon, France.

[13] Nantes Université, Inserm, CNRS, Université d'Angers, CRCI2NA, Nantes - France

[14] Department of Thoracic Oncology, Antwerp University Hospital, Edegem, Belgium.

[15] Clinic of Thoracic Oncology, Institut Jules Bordet, Université Libre de Bruxelles, Brussels, Belgium.

[#]These authors contributed equally to this work.

[§] S. Paternot (Sabine.Paternot@ulb.be) and P.P. Roger (Pierre.Roger@ulb.be) are co-corresponding authors.

***Address for all correspondence related to this submission to:** Pierre P. Roger, IRIBHM, Université Libre de Bruxelles, Campus Erasme, 808 route de Lennik, B-1070 Brussels, Belgium. Email: Pierre.Roger@ulb.be, Tel: +32 2 555 41 53, Fax: + 32 2 555 46 55.

This pdf file contains the supplementary references and figures (S1-S9).

Supplementary tables are in excel files:

One combined excel file for:

- **Table S1.** Drugs and antibodies
- **Table S2.** Cell lines characteristics
- **Table S3.** Quantification of viral genomes from RNA-seq data
- **Table S4.** Genes down- or up-regulated after long-term treatment with palbociclib (global analysis)
- **Table S6.** Differentially expressed pathways after long-term treatment with palbociclib in a global GSEA analysis: **A** down-regulated pathways, **B** up-regulated pathways
- **Table S8.** Tumors characteristics: **A** Clinical data, **B** RNA-seq data
- **Table S9.** Characteristics of the tumors from the TCGA-Meso cohort (**A**) and the Bueno et al. cohort (**B**)

One separate excel file for:

- **Table S5.** Genes down- or up-regulated after long-term treatment with palbociclib (analysis by cell line)
- **Table S7.** Pathways deregulated after long-term treatment with palbociclib in each cell line

Supplementary references

(1) Manfredi JJ, Dong J, Liu WJ, Resnick-Silverman L, Qiao R, Chahinian P, et al. Evidence against a role for SV40 in human mesothelioma. Cancer Res. 2005;65(7):2602-9. (cf. Table S2)

(2) Okonska A, Buhler S, Rao V, Ronner M, Blijlevens M, van der Meulen-Muileman IH, et al. Functional Genomic Screen in Mesothelioma Reveals that Loss of Function of BRCA1-Associated Protein 1 Induces Chemoresistance to Ribonucleotide Reductase Inhibition. Mol Cancer Ther. 2020;19(2):552-63. (cf. Table S2)

Supplementary Figure S1A

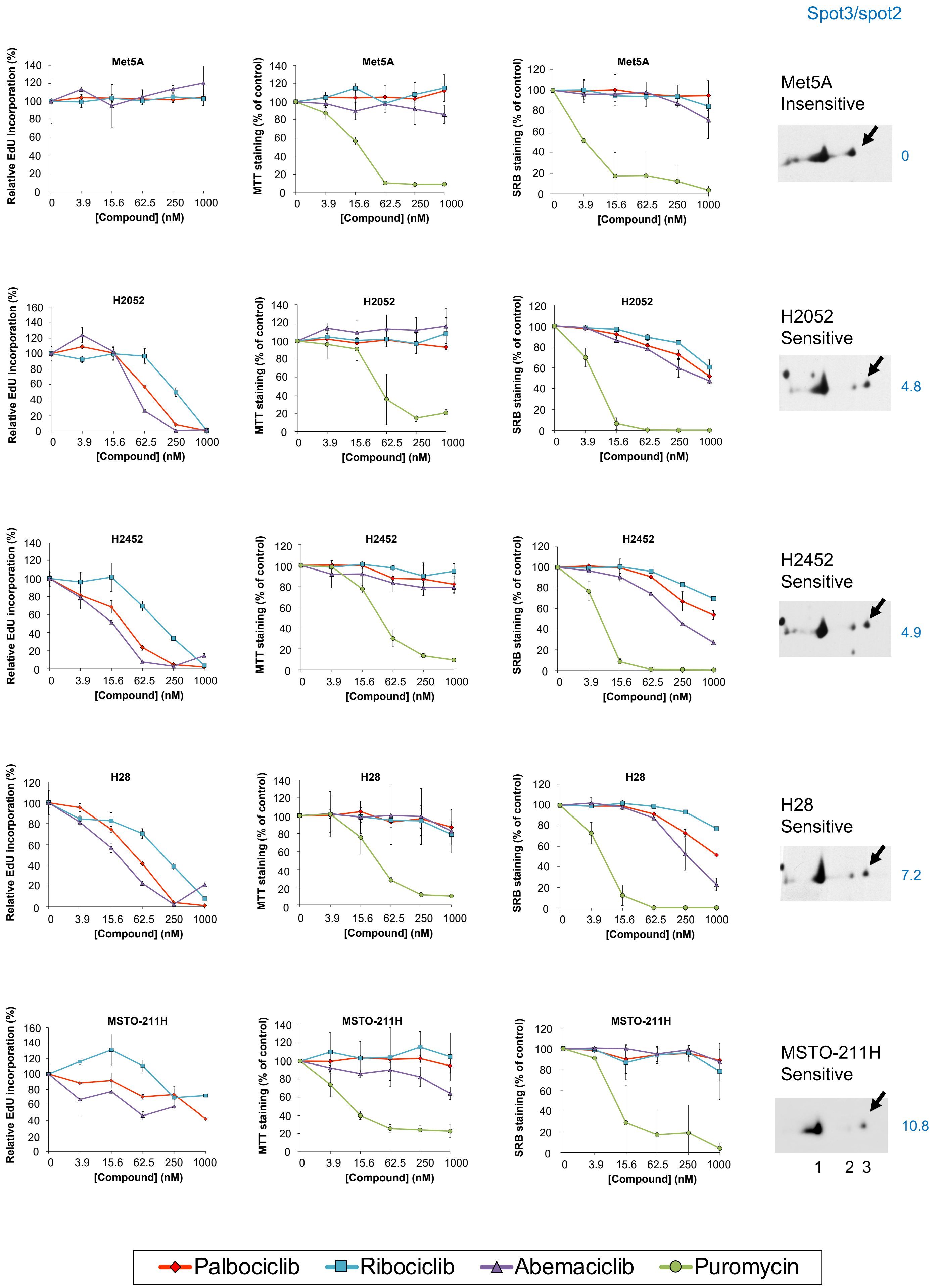

Supplementary Figure S1B

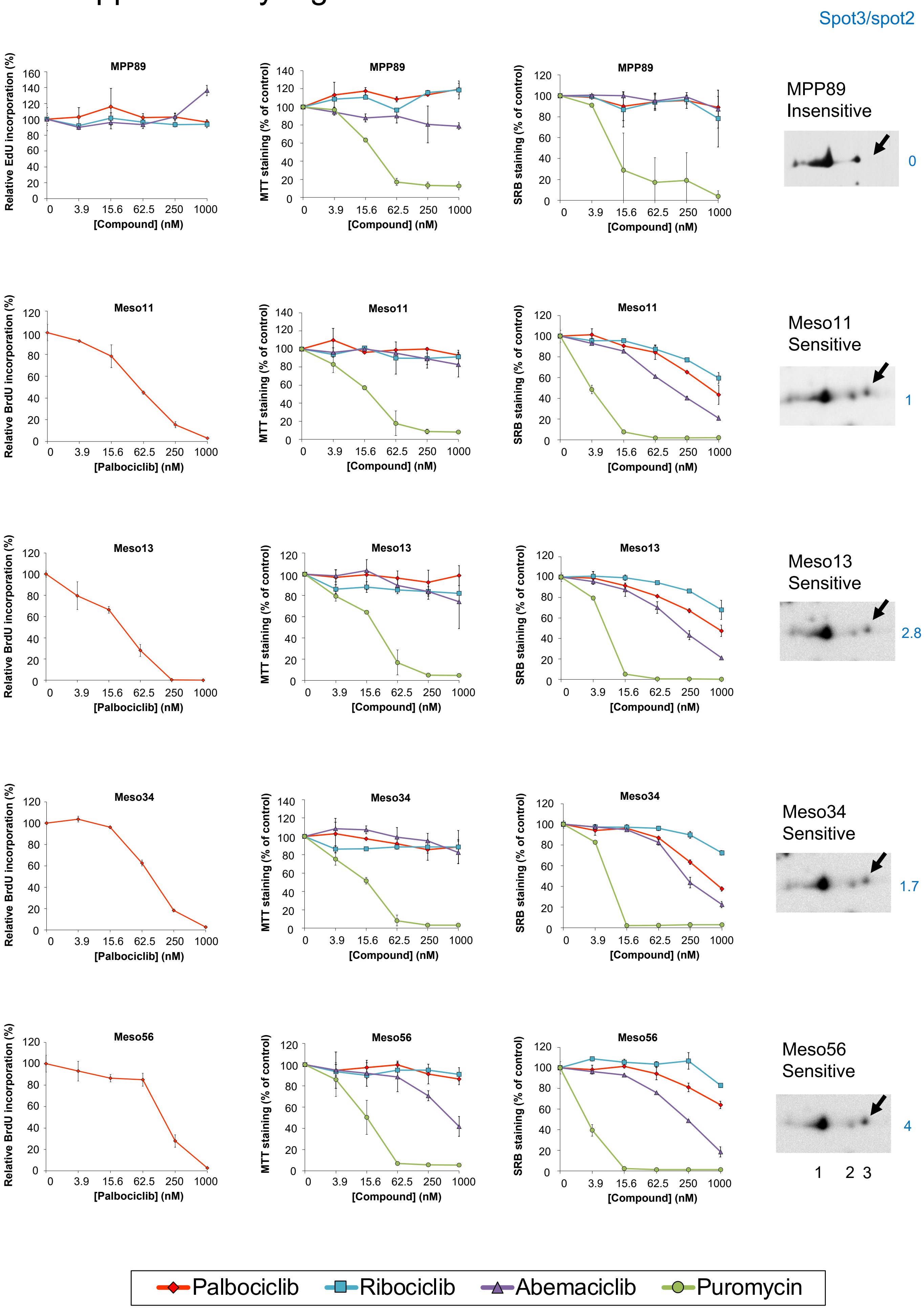

Supplementary Figure S1C

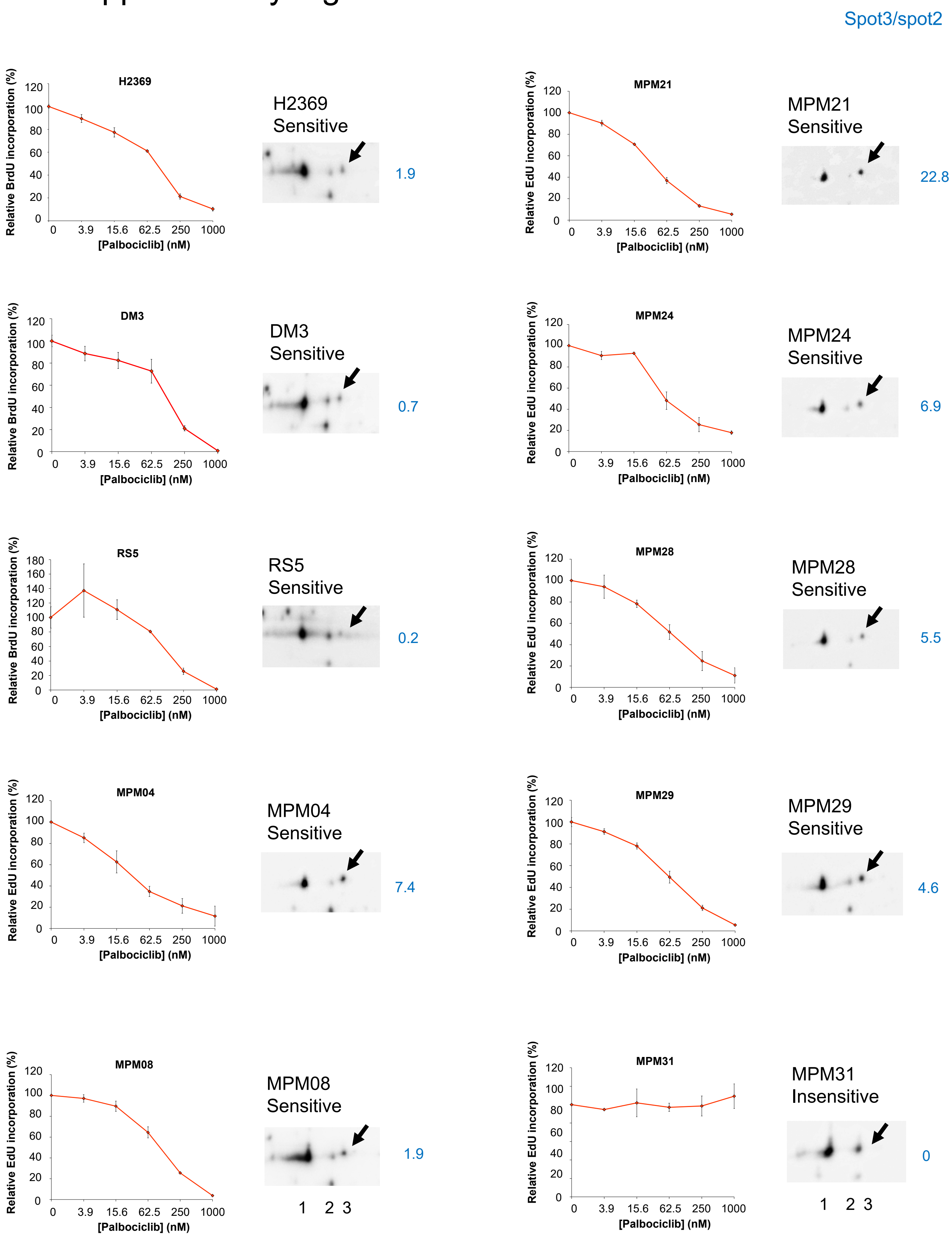

Supplementary Figure S1D

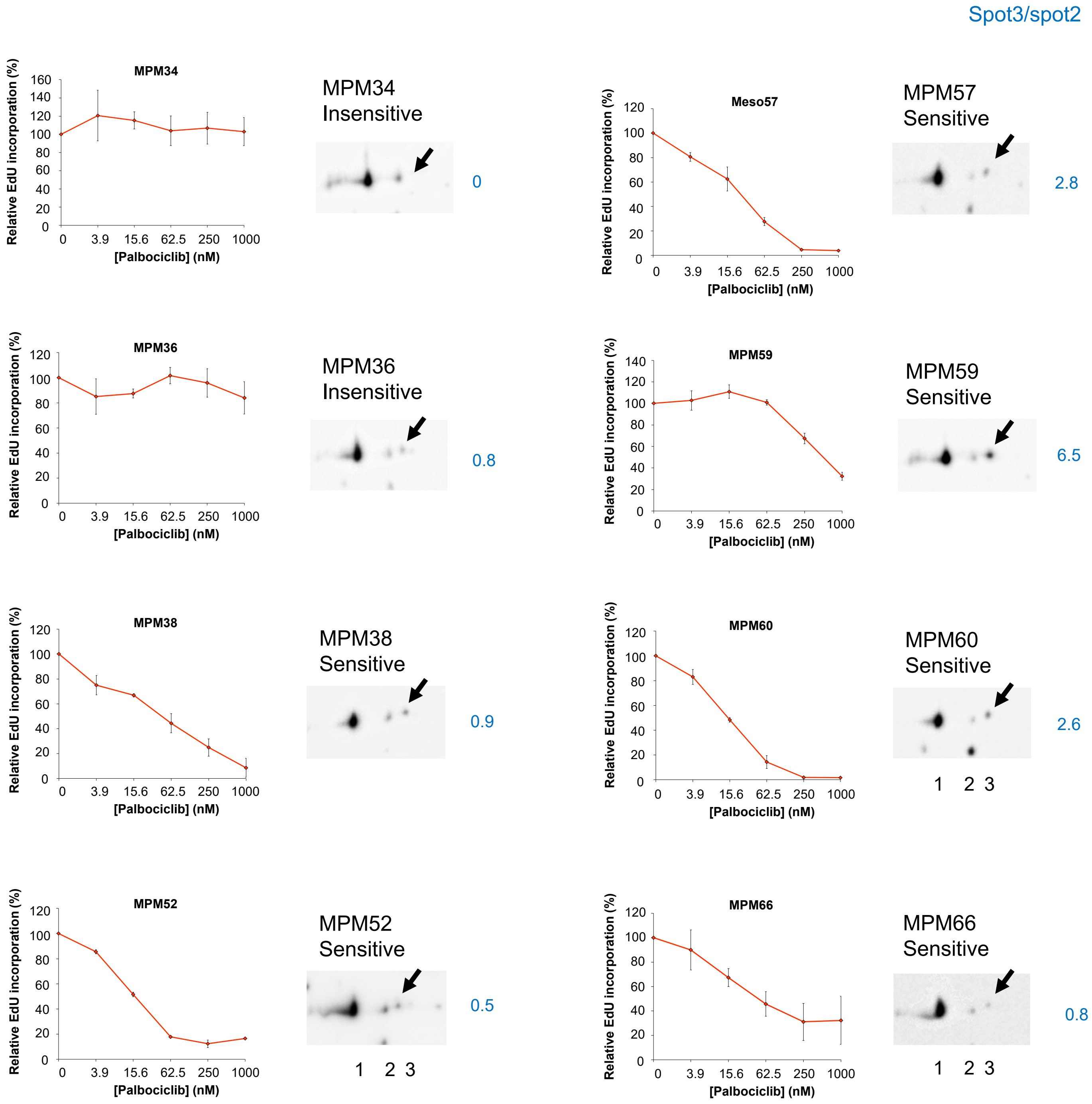

Supplementary Figure S1.
Sensitivity of MPM cell lines to palbociclib is associated with CDK4 phosphorylation.

Asynchronously growing MPM cell lines were treated for 24 h with DMSO, palbociclib, ribociclib or abemaciclib at the indicated concentrations. **A-D** DNA synthesis was evaluated from duplicated/triplicated dishes by counting the percentage of nuclei having incorporated BrdU/EdU during a pulse labeling of 1h. RS-5 cells were so slow growing that treatment with palbociclib was for 48 h with BrdU for the last 24 h. The relative proportion of BrdU/EdU-positive nuclei is expressed as percent of the mean value of control cells (**A-B** left panels, **C-D**). Error bars correspond to standard deviations scaled relative to the mean value of control cells except for the "MPM" cell lines (MPM_04 to MPM_66) where data represent mean +/- SD of 2 independent experiments. **A-B** The effect of CDK4/6 inhibitors was also measured after 48 h with MTT assay (middle panels) and after 6 days with the sulforhodamine assay (right panels). Increasing concentrations of puromycin were used as a positive control of cytotoxicity. Data represent mean +/- SD of 2 independent experiments. The profile of CDK4 separated by 2D-gel electrophoresis is also shown for each cell line. Arrows indicate the position of the T172 phosphorylated form of CDK4 (spot 3). The ratio of spot3/spot2 is displayed on the right of each panel.

A MeT-5A, H2052, H2452, H28, MSTO-211H

B MPP89, Meso11, Meso13, Meso34, Meso56

C H2369, DM-3, RS-5, MPM_04, MPM_08, MPM_21, MPM_24, MPM_28, MPM_29, MPM_31

D MPM_34, MPM_36, MPM_38, MPM_52, MPM_57, MPM_59, MPM_60, MPM_66

Supplementary Figure S2

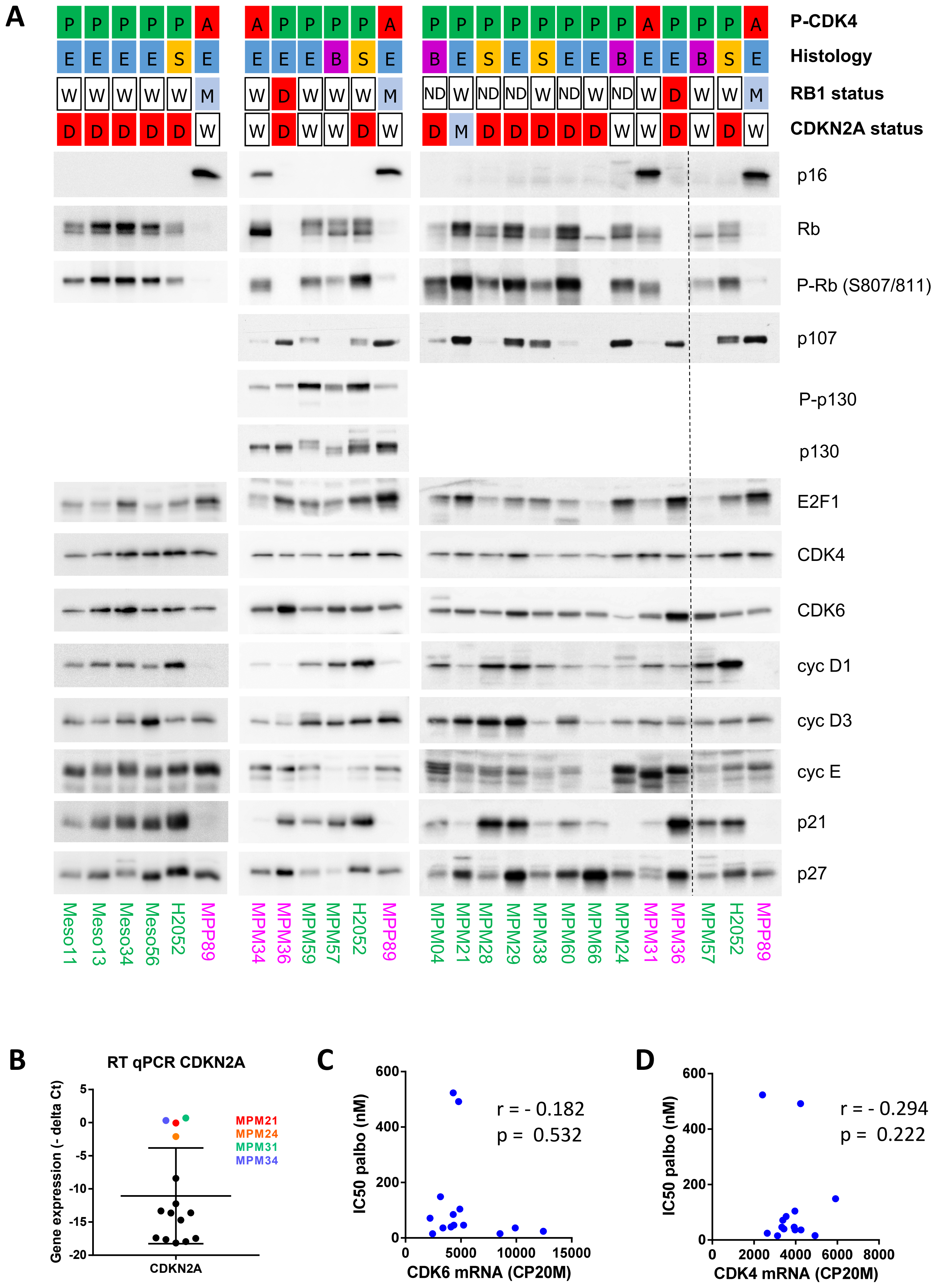

Supplementary Figure S2.
Absence of CDK4 phosphorylation in resistant cells is due to high p16 levels associated or not with pRb defect.

Sensitive cell lines are in green, resistant cell lines are in magenta.

A Total proteins were resolved by SDS-PAGE and detected with the indicated antibodies. The vertical dashed line separates parts of the same blot that were assembled. The following data are given for each cell lines: CDK4 phosphorylation profile (A: absent, P: phosphorylated), histology subtype (E: epithelioid, S: sarcomatoid, B: biphasic, N: normal), status of *RB1* and *CDKN2A* genomic locus (W: wild-type, D: deleted, M: mutated). The profile of CDK4 separated by 2D-gel electrophoresis is illustrated in Supplementary Figure S1.

B mRNA expression of *CDKN2A* measured by RT-qPCR in MPM_04 to MPM_66 cells.

C-D For sensitive cell lines, IC50 values for palbociclib as a function of *CDK6* (**C**) or *CDK4* (**D**) mRNA levels (RNA-seq data in CP20M). Correlation between IC50 and *CDK4* or *CDK6* expression was evaluated with a Spearman's rank test.

Supplementary Figure S3

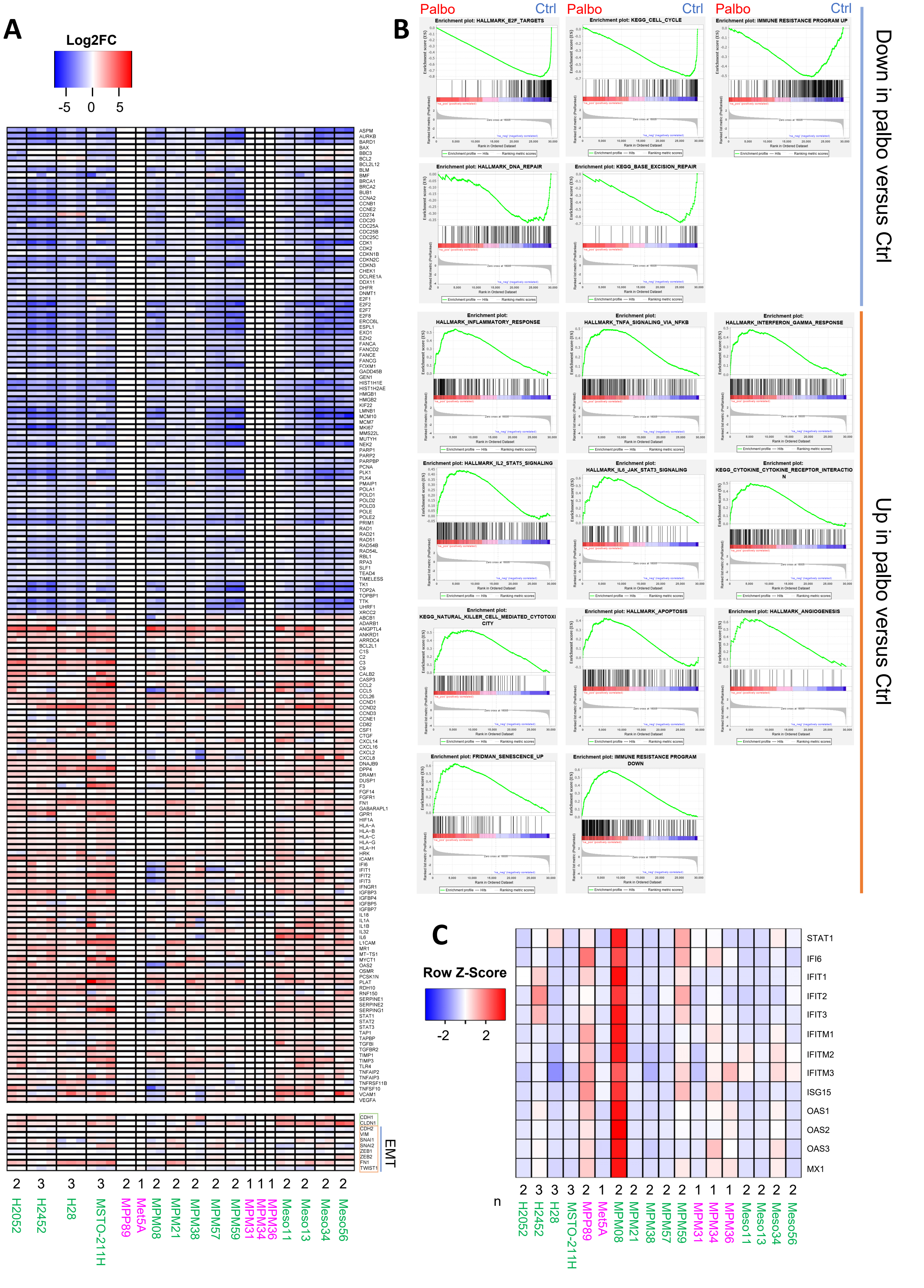

Supplementary Figure S3.
Impact of long term treatment with palbociclib analyzed by RNA-seq.

A Heatmap representing genes down- or up-regulated after a treatment of 9-10 days with 1 µM palbociclib. Genes involved in epithelial-mesenchymal transition (EMT) are grouped at the bottom of the plot (orange frame). Green frame: epithelial markers. Data are presented as log2FC (palbo/ctrl). n independent experiments are shown, as stated below the heatmap. Sensitive cell lines are in green, resistant ones are in magenta.

B GSEA enrichment plots for selected pathways down- or up-regulated by 1 µM palbociclib relative to control in a global analysis using all sensitive cell lines (n=13).

C Heatmap representing basal expression of genes involved in the Interferon pathways. Data are presented as mean of n independent experiments, as stated below the heatmap. Sensitive cell lines are in green, resistant ones are in magenta.

Supplementary Figure S4

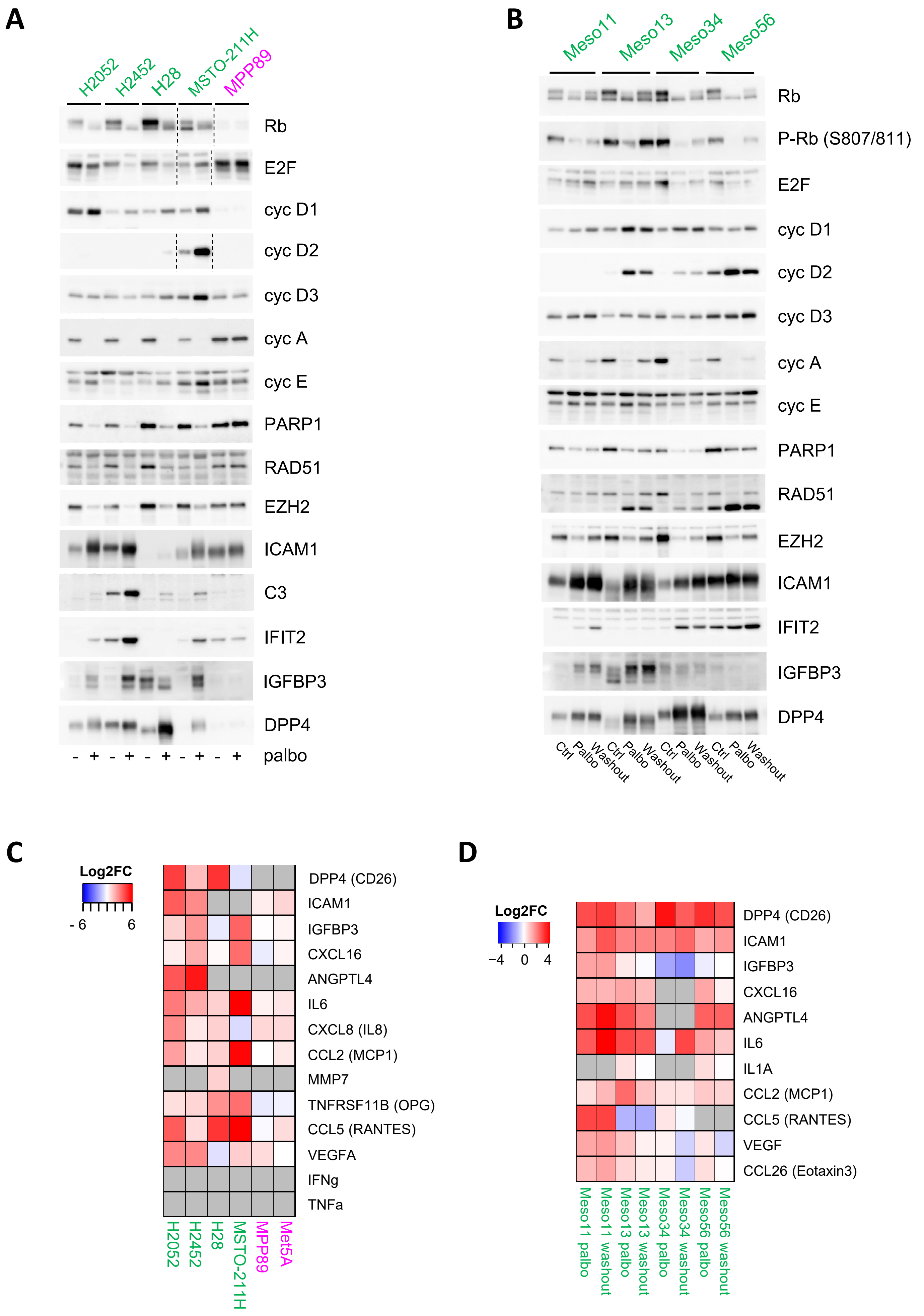

Supplementary Figure S4.
Validation of RNA-seq data at the protein level.

Cells were treated for 9-10 days with DMSO (Ctrl) or palbociclib (Palbo) at 1 µM. In **B** and **D**, a drug washout of 48 h was also performed. Sensitive cell lines are in green, resistant ones are in magenta.

A-B Western Blot analysis with the indicated antibodies.

C-D Quantification of the indicated secreted proteins from cell culture supernatants by multiplex Elisa array. Conditioned media were collected 72 h (**C**) or 48 h (**D**) after the last change of medium. Heatmap represents log2FC (palbo/ctrl and washout/ctrl). Values under the detection limit are in grey.

Supplementary Figure S5A

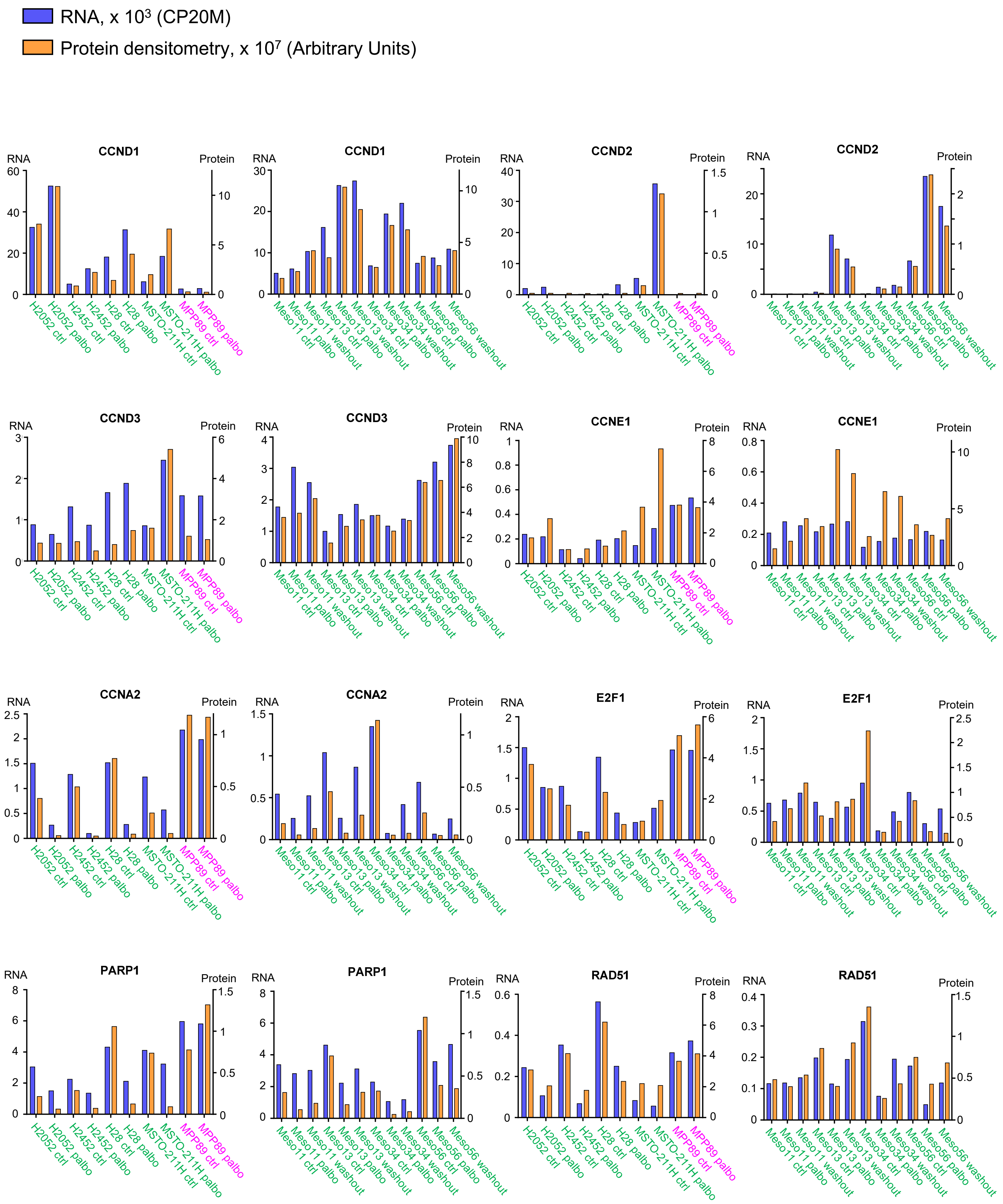

Supplementary Figure S5B

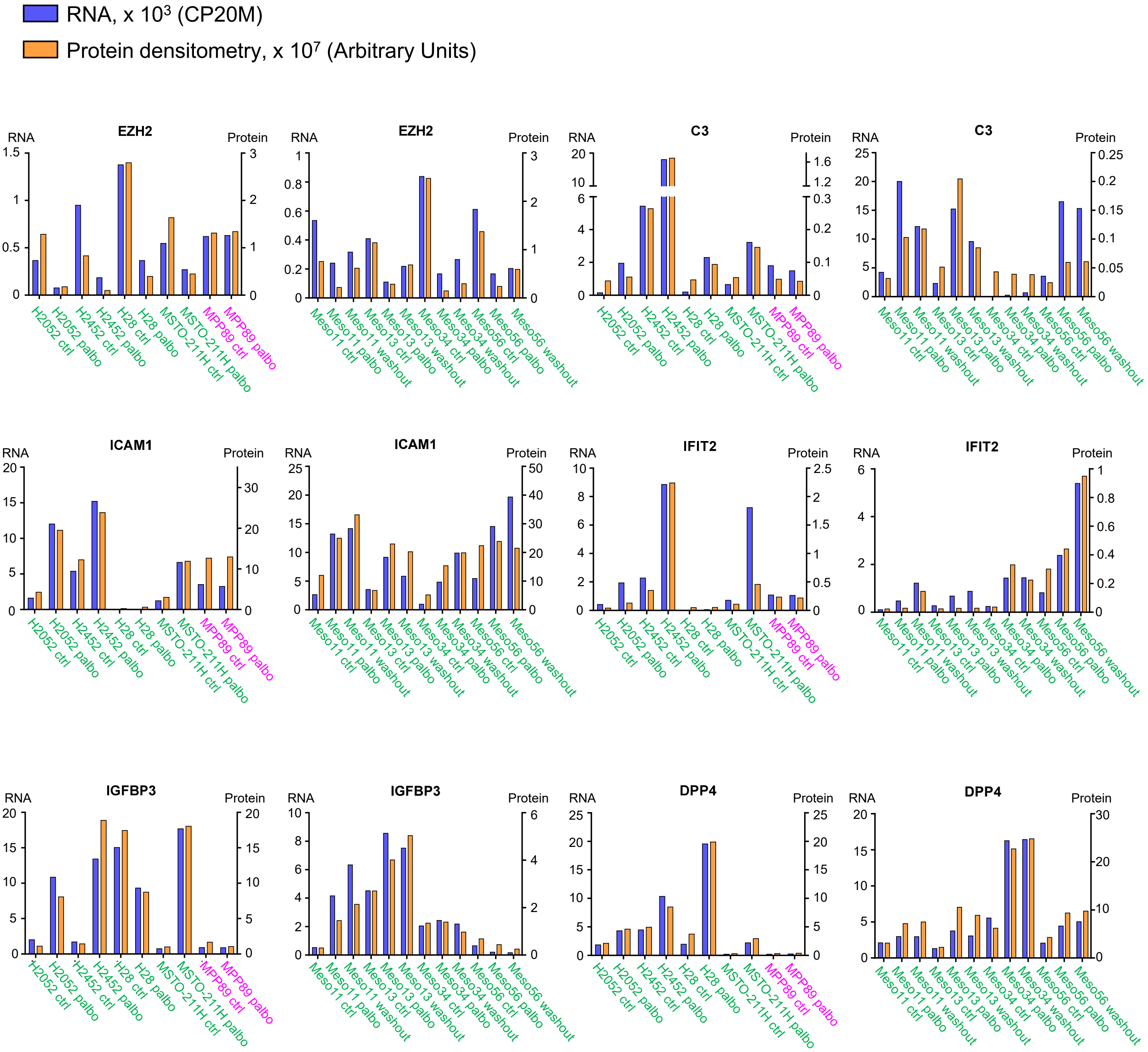

Supplementary Figure S5C

RNA, x 10^3 (CP20M)

Protein concentration in cell culture supernatant, x 10^3 (pg/ml)

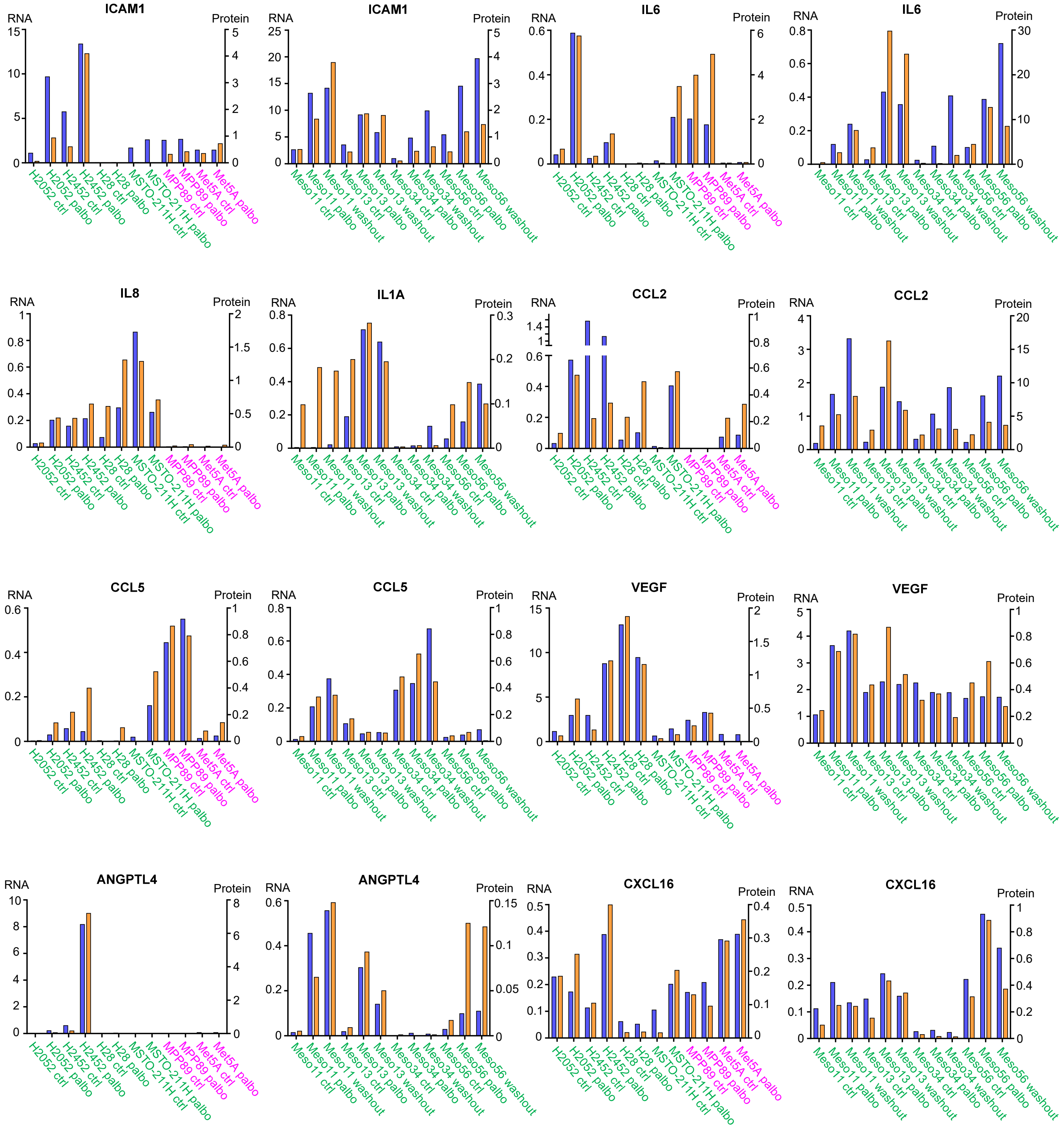

Supplementary Figure S5D

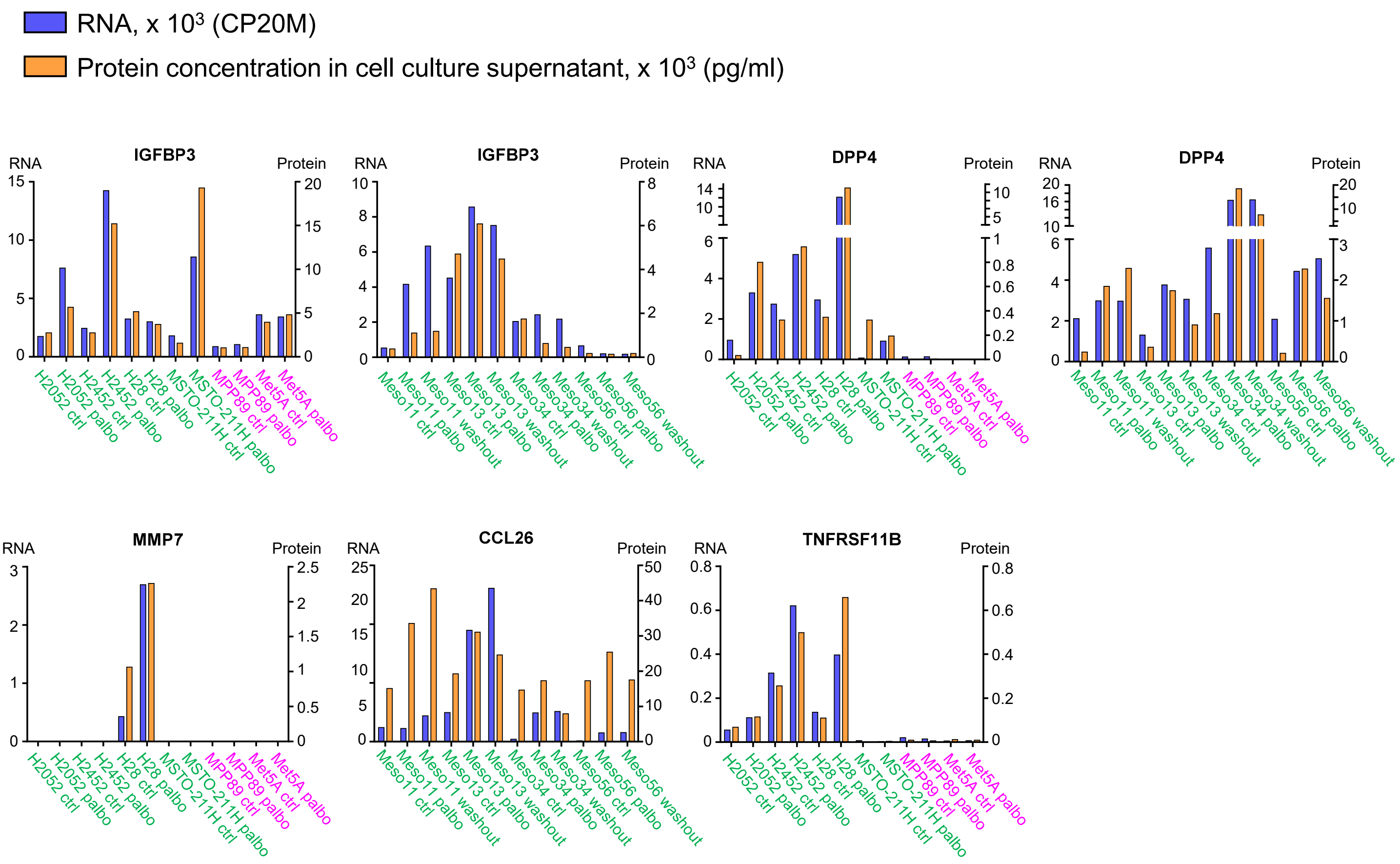

Supplementary Figure S5.
Concordance between RNA-seq data and proteic analyses.

Sensitive cell lines are in green, resistant cell lines are in magenta.

A-B Plots combining, for indicated genes, mRNA levels from RNA-seq data normalized to the library size (CP20M) and the quantification of the corresponding protein from immunodetections.

C-D Plots combining, for indicated genes, mRNA levels from normalized RNA-seq data (CP20M) and the quantification of the corresponding protein from culture supernatants by multiplex Elisa.

Supplementary Figure S6

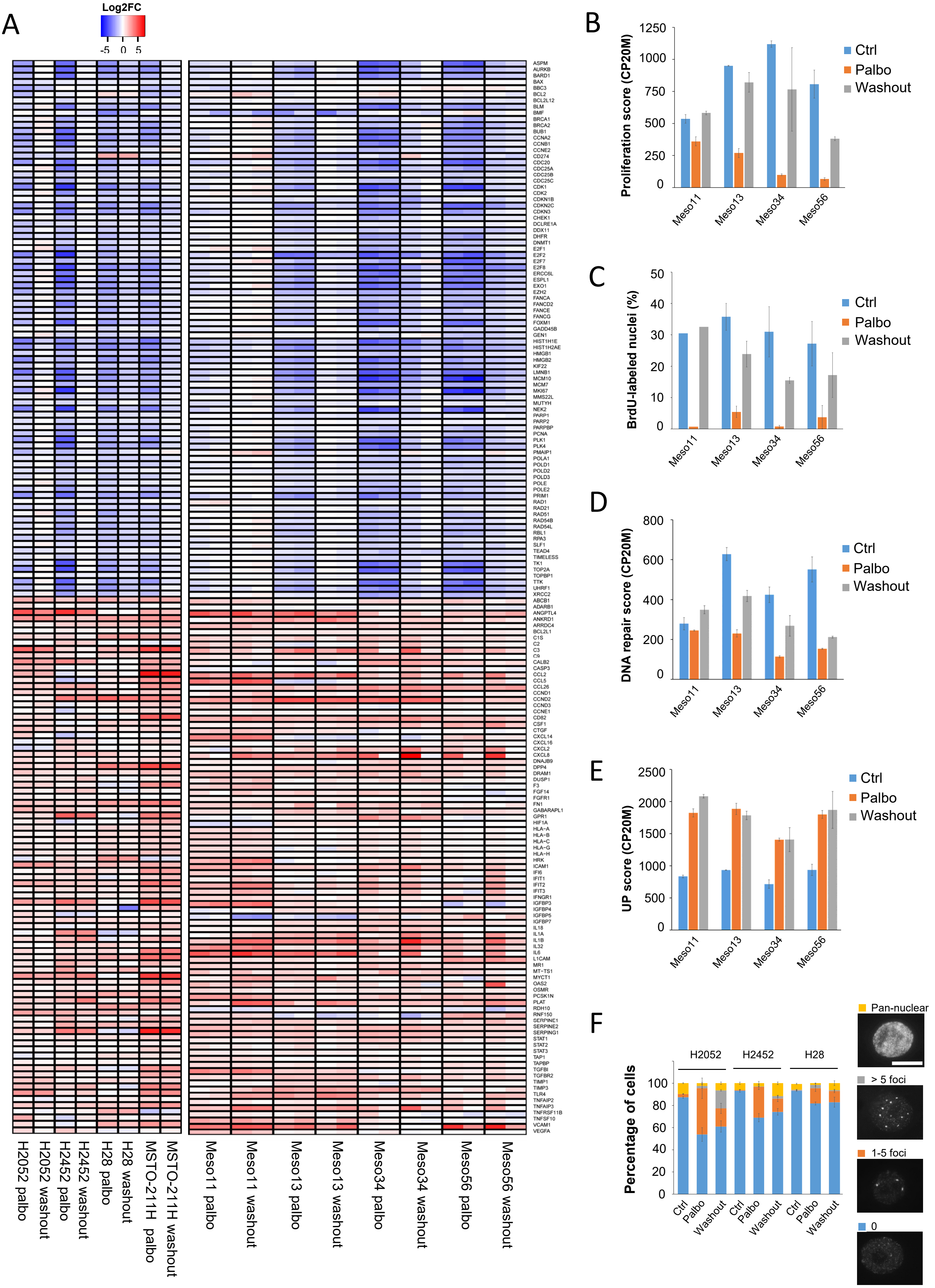

Supplementary Figure S6.
Effect of palbociclib washout.

A Cells were treated for 10 days with 1 µM palbociclib followed or not by a drug washout of 48 h. Heatmap representing genes down- or up-regulated by palbociclib and the impact of drug washout on their expression. Data are presented as log2FC (palbo/ctrl and washout/ctrl). n independent experiments are shown, as stated below the heatmap.
B Proliferation score calculated from the RNA-seq data illustrated in **A** and expressed in counts per 20 million reads (CP20M). Mean +/- SEM of 2 independent experiments.
C DNA synthesis in cells treated as in **A**. Mean +/- SEM of 3 independent experiments except for meso11 (n=1).
D DNA repair score calculated from the RNA-seq data illustrated in **A** and expressed in counts per 20 million reads (CP20M). This score represents the median expression of the genes involved in DNA repair illustrated in Figure 5B. Mean +/- SEM of 2 independent experiments.
E Expression score of up-regulated genes calculated from the RNA-seq data illustrated in **A** and expressed in counts per 20 million reads (CP20M). Mean +/- SEM of 2 independent experiments.
F Percentage of nuclei displaying a pan-nuclear γH2AX staining, from 1 to 5 foci or more than 5 γH2AX foci in cells treated as in **A**. Mean +/- SEM of 2 independent experiments. Representative images are shown on the right. Scale bars, 20 µM.

Supplementary Figure S7

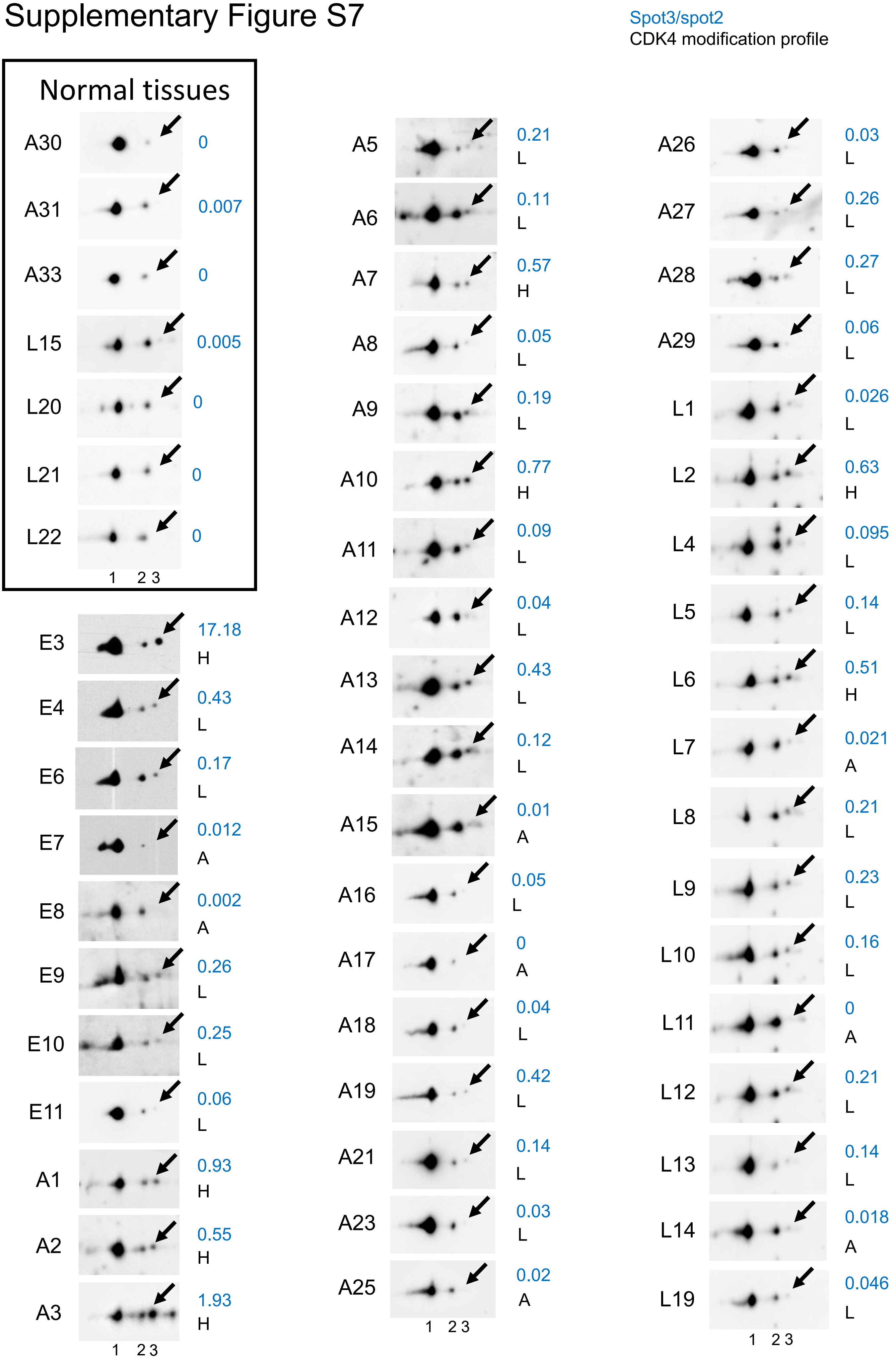

Supplementary Figure S7.
CDK4 phosphorylation is present in most MPM tumors.

CDK4 immunodetection after separation by 2D-gel electrophoresis from 7 normal pleurae and 47 mesotheliomas. Arrows indicate the position of the T172 phosphorylated form of CDK4 (spot 3). Quantification of phosphorylated CDK4 (spot3/spot2) is indicated on the right and the CDK4 modification profile (H, L or A) is indicated for each tumor.

Supplementary Figure S8

A

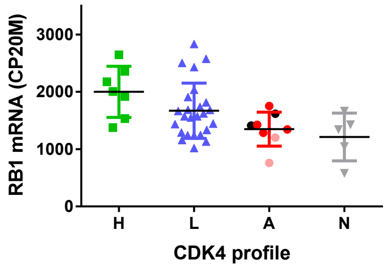

B C D E

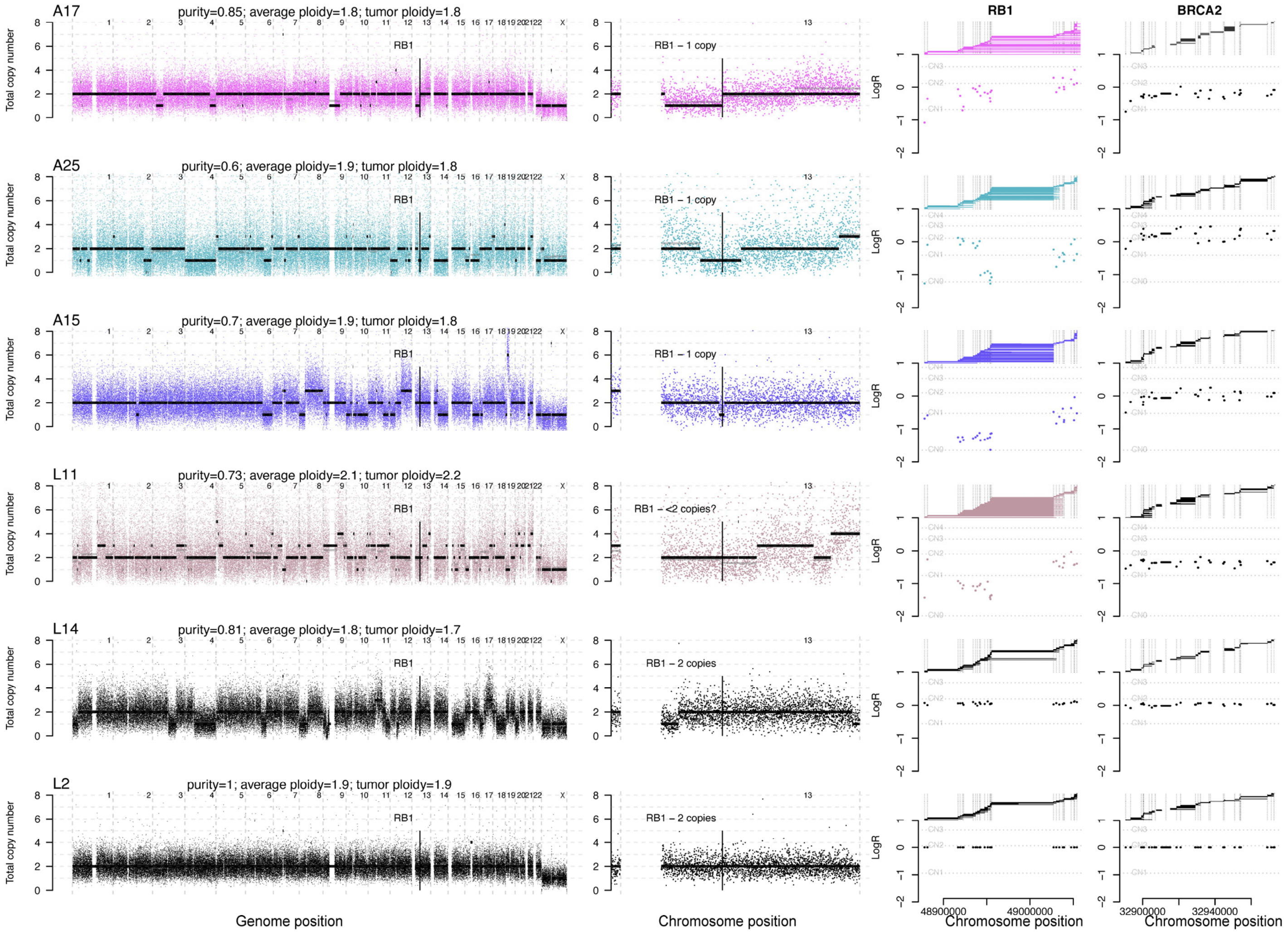

Supplementary Figure S8.
Double hits in *RB1* in high p16 expressing tumors: loss-of-heterozygosity and aberrant splicing.

A *RB1* mRNA levels (RNA-seq data in CP20M) in the different classes of tumors and in normal tissues (N). n=7 for profile H, n=25 for profile L, n=8 for profile A and n=5 for normal pleura. For class A tumors: red dots = absence of CDK4 phosphorylation with high *CDKN2A* expression and high proliferation score, pink dots = absence of CDK4 phosphorylation with low *CDKN2A* expression and high proliferation score, black dots = absence of CDK4 phosphorylation with intermediate *CDKN2A* expression and low proliferation score. Error bars: mean +/- SD. No significant difference between groups was found using the Kruskal-Wallis test. **B-E** DNA-sequencing was performed for the four class A tumors with high p16 expression (A17, A25, A15 and L11). L14 and L2 tumors were used as controls.

B Genome-wide total copy-number profile across chromosomes. The raw underlying data is shown for each 30kb bins, the raw segment average (in gray) and the fitted rounded integer value (in black). The position of *RB1* is also shown.

C Zoom in chromosome 13 showing the number of copies of *RB1*.

D On the bottom graph, we show the logged read-depth ratios of the exon targets of *RB1* in the targeted DNA-sequencing data (y-axis) mapped to their genomic coordinates (x-axis). Horizontal lines show where the logged read-depth ratios would fall for different numbers of DNA copies, estimated from the sample purity and ploidy (Methods). On top of this graph, for each RNA-sequenced read that is mapped to an exon junction and sorted by its mapping position, we link it to its mate position. Horizontal lines therefore correspond to the junctions between exons like in a Sashimi plot. Vertical dotted lines indicate the position of the exons.

E Same as in **D** but for a control gene *BRCA2*.

Supplementary Figure S9

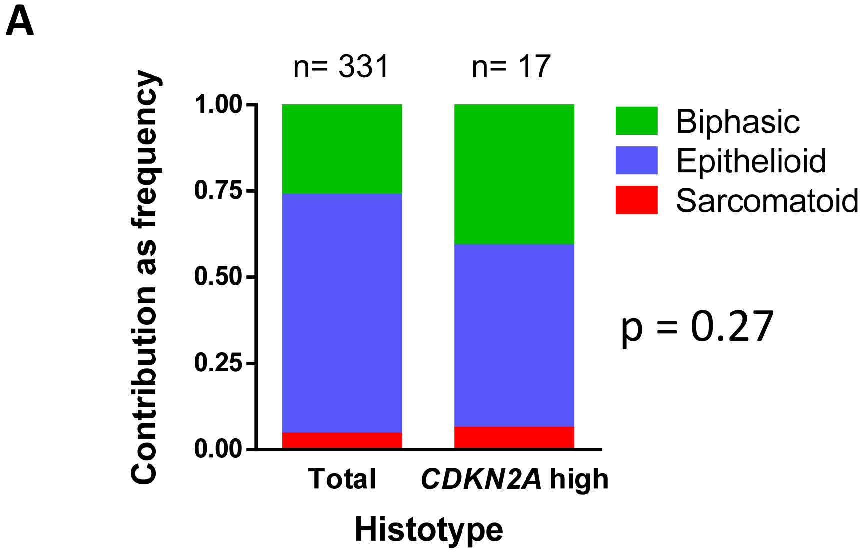

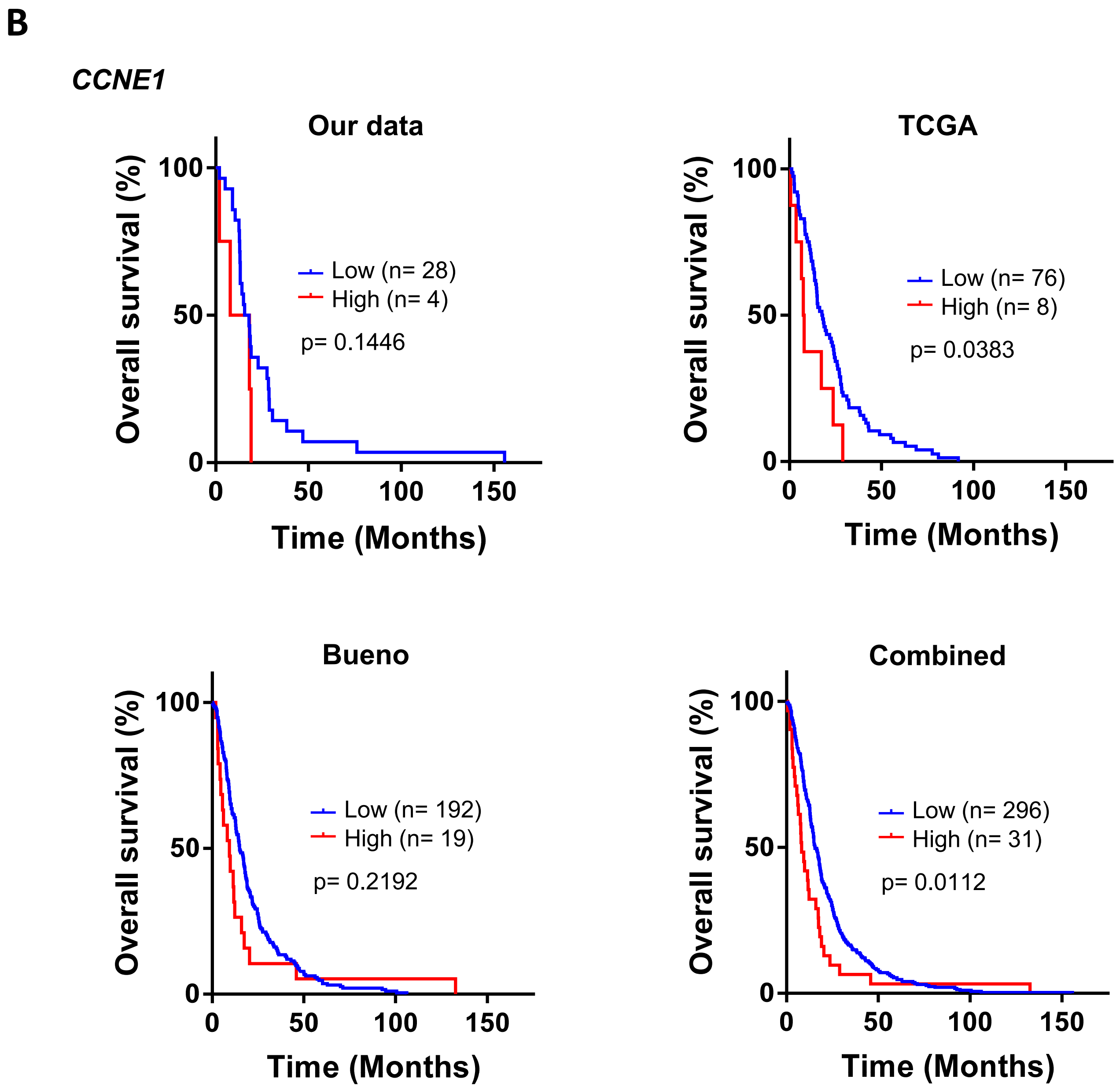

Supplementary Figure S9.
High expression of *CCNE1* is associated with a shorter overall survival in MPM patients.

A Relative proportions of the three MPM histotypes in the *CDKN2A* high tumors versus all tumors of the combined cohort. The number of samples in each series is indicated at the top of each bar. p-value, Fisher's Exact Test.
B Kaplan-Meier curves comparing survival of patients with high or low *CCNE1* expression in our data (n=32), the TCGA (n=84) and Bueno et al. (n=211) cohorts; or in the combination of the three cohorts (n=327). P-values, log-rank test.
